# Attractor Landscape Analysis Reveals a Reversion Switch in the Transition of Colorectal Tumorigenesis

**DOI:** 10.1002/advs.202412503

**Published:** 2025-01-22

**Authors:** Dongkwan Shin, Jeong‐Ryeol Gong, Seoyoon D. Jeong, Youngwon Cho, Hwang‐Phill Kim, Tae‐You Kim, Kwang‐Hyun Cho

**Affiliations:** ^1^ Department of Bio and Brain Engineering Korea Advanced Institute of Science and Technology (KAIST) Daejeon 34141 Republic of Korea; ^2^ Research Institute National Cancer Center Goyang 10408 Republic of Korea; ^3^ Department of Cancer Biomedical Science National Cancer Center Graduate School of Cancer Science and Policy Goyang 10408 Republic of Korea; ^4^ Department of Molecular Medicine and Biopharmaceutical Sciences Graduate School of Convergence Science and Technology Seoul National University Seoul 03080 Republic of Korea

**Keywords:** attractor landscape analysis, cancer reversion, colon cancer, critical transition, dynamic network model, patient‐derived organoid, single cell transcriptome data

## Abstract

A cell fate change such as tumorigenesis incurs critical transition. It remains a longstanding challenge whether the underlying mechanism can be unraveled and a molecular switch that can reverse such transition is found. Here a systems framework, REVERT, is presented with which can reconstruct the core molecular regulatory network model and a reversion switch based on single‐cell transcriptome data over the transition process is identified. The usefulness of REVERT is demonstrated by applying it to single‐cell transcriptome of patient‐derived matched organoids of colon cancer and normal colon. REVERT is a generic framework that can be applied to investigate various cell fate transition phenomena.

## Graphical abstract

REVERT (REVERse Transition) is presented, a systems framework developed to identify a reversion switch in the transition process of tumorigenesis through comprehensive attractor landscape analysis of single‐cell transcriptome data. The longstanding challenge of understanding the critical transition during cell fate change and revealing a hidden reversion switch is addressed, with a particular focus on colorectal tumorigenesis.

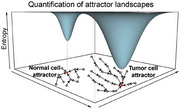

## 1 Introduction

Cell fate changes often involve abrupt transition, called “critical transition,”^[^ ^1^ ^]^ at key points, superimposed on a background of more gradual changes. In particular, it has been well known that tumorigenesis incurs such critical transition.^[^ ^1^, ^2^ ^]^ So, questions arise as to what the core molecular regulatory network underlying the critical transition is and whether we can reverse it by controlling a master regulator of the core network. Among the cell fate changes, cancer reversion refers to reprogramming of cancer cells into normal (or normal‐like) cells which exhibit normal phenotypes and also lose malignant characteristics. Since Askanazy's historic observation of spontaneous reversion of ovarian teratomas within the embryonic microenvironment,^[^ ^3^ ^]^ a number of intriguing studies have been followed to the present reporting the possibility of reverting cancer cell states to phenotypically healthy cell states under various experimental settings.^[^ ^4^ ^]^ However, these approaches often relied on trial‐and‐error experiments or comparative analyses mostly that focus on static network properties, limiting their ability to capture dynamic transitions. Recent studies aimed to overcome such limitations by developing mechanistic molecular regulatory network models for predicting master regulators of cell‐fate reprogramming in breast^[^ ^5^ ^]^ and lung cancers.^[^ ^6^ ^]^ However, these approaches are limited by their reliance on literature‐based models, which are often incomplete and biased toward well‐studied genes like oncogenes or tumor suppressors.

Recently, the rapid growth of single‐cell transcriptomic data provides unprecedented opportunities to unravel cellular heterogeneity within tissues and investigate dynamic cellular processes such as cell state transitions during differentiation, development, or epithelial‐mesenchymal transition.^[^ ^7^ ^]^ In particular, pseudotime analysis allows us to explore transition states or intermediate cell states between distinct cellular states, facilitating the identification of transition genes, a set of genes that switch their expressions during the transition.^[^ ^8^ ^]^ This analysis also aids in the inference of static gene regulatory networks for each cell state or a specific pseudotime trajectory and also the reconstruction of mechanistic models for molecular regulation networks based on ordinary differentiation equations (Inference Snapshot,^[^ ^9^ ^]^ SCODE,^[^ ^10^ ^]^ SCOUP^[^ ^11^ ^]^) or Boolean functions (Pseudotime‐network‐inference,^[^ ^12^ ^]^ BTR,^[^ ^13^ ^]^ SCNS,^[^ ^14^ ^]^ IQCELL^[^ ^15^ ^]^), which can elucidate the dynamic changes of gene expression along the pseudotime trajectory (see Review^[^ ^16^ ^]^). Of note, these dynamic network models allow us not only to investigate the fundamental mechanisms governing cell fate transitions during development or disease progression but also to predict dynamic responses to gene perturbations based on the static single‐cell transcriptome data. Recent advances in gene regulatory network modeling have offered insights into controlling cell fates, but modeling tumorigenesis remains challenging due to genetic alterations that dynamically reshape networks throughout the tumorigenic process, hindering the creation of a unified model. Moreover, critical transitions often observed in many biological state transitions such as tumorigenesis add another challenge owing to the nature of rapid change in phenotypes occurring at a tipping point.^[^ ^1^, ^2^ ^]^ This critical transition is actually a key feature of tumorigenesis and thereby understanding the underlying complex dynamics at the tipping point is crucial for exploring cancer reversion.^[^ ^4e^ ^]^ Instead of modeling the entire tumorigenic trajectory, focusing on the tipping point captures key state transitions.

**Figure 1:**
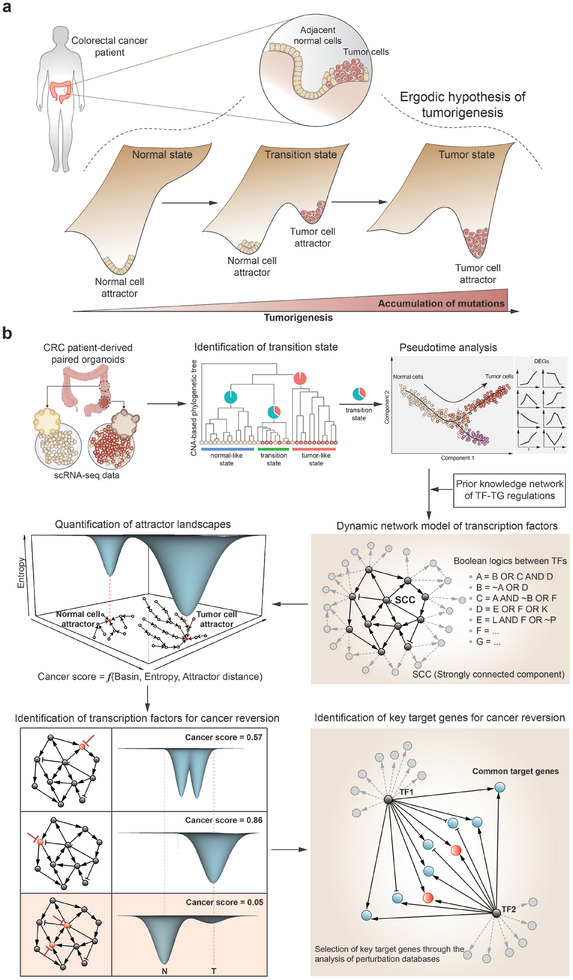
Conceptual framework of tumor transition state and overview of REVERT. a) Conceptual framework of tumor transition state. The ergodic hypothesis in statistical physics suggests that a snapshot of a cell population taken from a cancer patient would capture the entire process of tumorigenesis. This includes the normal state with a stable normal cell attractor in an epigenetic landscape, the intermediate transition state in which the normal cell attractor destabilizes while a tumor cell attractor emerges and stabilizes, and the eventual tumor state where the tumor cell attractor dominates. b) Workflow of REVERT.

Recent studies showed that cell fate changes typically occur between discrete steady states, known as attractor states, and heterogeneity in gene expression is observed as cells transition between these states.^[^ ^7^, ^17^ ^]^ The collection of heterogeneous transcriptional profiles observed during a cell fate change represents a ‘transition state’ where a cell exhibits a mixed identity between two or more states. A number of attempts have been made to identify such transition states and relevant marker genes in the process of cell fate changes such as epithelial‐mesenchymal transition (EMT), differentiation, and development. They are mostly based on the heterogeneity or plasticity of transition states, and include those methods like Index of criticality (Ic),^[^ ^1a^ ^]^ QuanTC,^[^ ^1e^ ^]^ scRCMF,^[^ ^8c^ ^]^ MuTrans,^[^ ^8d^ ^]^ and BioTIP.^[^ ^8b^ ^]^ In tumorigenesis, normal cells in a stable state (normal cell attractor) within the attractor landscape become destabilized due to accumulating genetic alterations, transitioning into a bistable state between normal and tumor cell attractors (**Figure** **1**a). This tumor transition state is unstable and reversible, allowing cells to stochastically switch between the two attractors. Despite sharing the same gene regulatory network structure due to a common mutational background, cells in this state exhibit diverse gene expression profiles along the transition trajectory. This heterogeneity is crucial for developing a mechanistic model of the core regulatory network driving tumorigenesis, as it reflects the dynamic changes occurring within a single network framework.

Here, we present REVERT (REVERse Transition), a novel systems framework to identify reversion switches in the transition of tumorigenesis through attractor landscape analysis. REVERT uses single‐cell transcriptomic data of cancer patients as an input and employs a Boolean network modeling approach to develop dynamic gene regulatory network models over the transition path from normal to cancer cell attractors within the tumor transition state, where cell states are represented by the network states dynamically changing upon a single backbone of the gene regulatory network (GRN). The Boolean model of GRN offers a comprehensive view of the relationship among network states, termed an attractor landscape. By introducing a malignancy score quantifying the attractor landscape of the transition state, REVERT can estimate the malignant potency of the cellular state based on the concept of Waddington's landscape of cancer and identify an optimal therapeutic intervention target which minimizes the score. By applying it to single‐cell transcriptome of colorectal cancer patient‐derived tumor and adjacent normal organoids, REVERT identifies combinations of transcription factor targets for cancer reversion through systematic in silico perturbation analysis. We further validate the predicted restoration effect of normal phenotypes through experiments with colorectal cancer organoids by controlling the reversion switch, USP7, a common target gene among the identified transcriptional factors. Together, these results highlight the usefulness of REVERT in identifying optimal therapeutic targets and provide valuable insights into potential cancer reversion strategies. REVERT can be widely applied to identify optimal reprogramming targets for desired cell fate changes in differentiation, development, or cancer cell reprogramming or reversion, while offering mechanistic insights into dynamic gene regulation underlying various cell state transitions.

## 2 Results

### 2.1 Overview of REVERT

REVERT aims to reconstruct the dynamic network model of GRN that can represent cellular dynamics in the tumor transition state and to identify potential intervention targets for cancer reversion through in silico perturbation simulations with attractor landscape analysis. Dynamic network modeling is crucial for understanding and predicting the behavior of a system in response to perturbations under untested circumstances, enabling the identification of an optimal reversion switch that can drive the cellular system toward a desired normal cell state upon the attractor landscape. To ensure the inclusion of a sufficient number of cells in the transition state and also in the normal and cancer cell states representing the endpoints of tumorigenesis, REVERT uses paired single‐cell transcriptomic data from tumor and adjacent normal tissues as input. This approach is based on the ergodic hypothesis in statistical physics,^[^ ^18^ ^]^ which means that a snapshot of cell population at a single sampling time point can actually represent all possible intermediate cellular states along the normal‐to‐tumor cell state transition trajectory (**Figure** **1**a).

REVERT consists of four main steps (Figure 1b): i) Identification of a transition state based on copy number variants (CNVs) of tumor and matched normal tissues. For this purpose, we employ an algorithm, such as CopyKAT,^[^ ^19^ ^]^ to infer single‐cell CNV profiles from scRNA‐seq data, as it still remains challenging to perform sequencing of both the genome and the transcriptome at the same time from a same single cell. We cluster the inferred CNV patterns to deduce the evolutionary trajectory of the tumor, which is typically described by a phylogenetic tree. This allows for the identification of distinct subclones, including a normal‐like, tumor‐like, and transition states characterized by the coexistence of both normal and tumor cells. ii) Reconstruction of a dynamic network model to capture the dynamic behavior of transition cells. There may be various stochastic transitions occurring between steady states corresponding to normal and tumor phenotypes within a transition state.^[^ ^4^, ^7^ ^]^ By employing pseudotime analysis on the transition state, we obtain the temporal progression of differentially expressed genes (DEGs) along the trajectory from a normal cell state to a tumor cell state. By integrating the temporal information of DEGs with prior knowledge about regulatory interactions between genes, we construct Boolean logic functions that describe the regulatory relationships between transcriptional factors (TFs) within a strongly connected component (SCC), a subgraph composed of feedback structures where any two nodes are mutually reachable. iii) Introduction of a cancer score (CS) that quantifies an attractor landscape, which comprises the normal or tumor cell attractor states arising from the Boolean network dynamics, by measuring the relative dominance and stability of normal and tumor attractors in the state transition graph. iv) Identification of target TFs for cancer reversion by investigating cancer score changes in response to in silico knockout or overexpression of TFs. The optimal combination of TFs is filtered based on the resulting cancer score, with a lower CS indicating a closer proximity to the normal cell state. Perturbing candidate TFs in the SCC affects all target genes within the GRN through the propagation of the signal flow from TFs to their respective target genes. We further refine key target genes among the common target genes of the candidate TFs by analyzing perturbation databases such as LINCS^[^ ^20^ ^]^ (http://www.lincsproject.org/) and DepMap^[^ ^21^ ^]^ (https://depmap.org/portal/).

### 2.2 Identifying the Tumor Transition State

**Figure 2:**
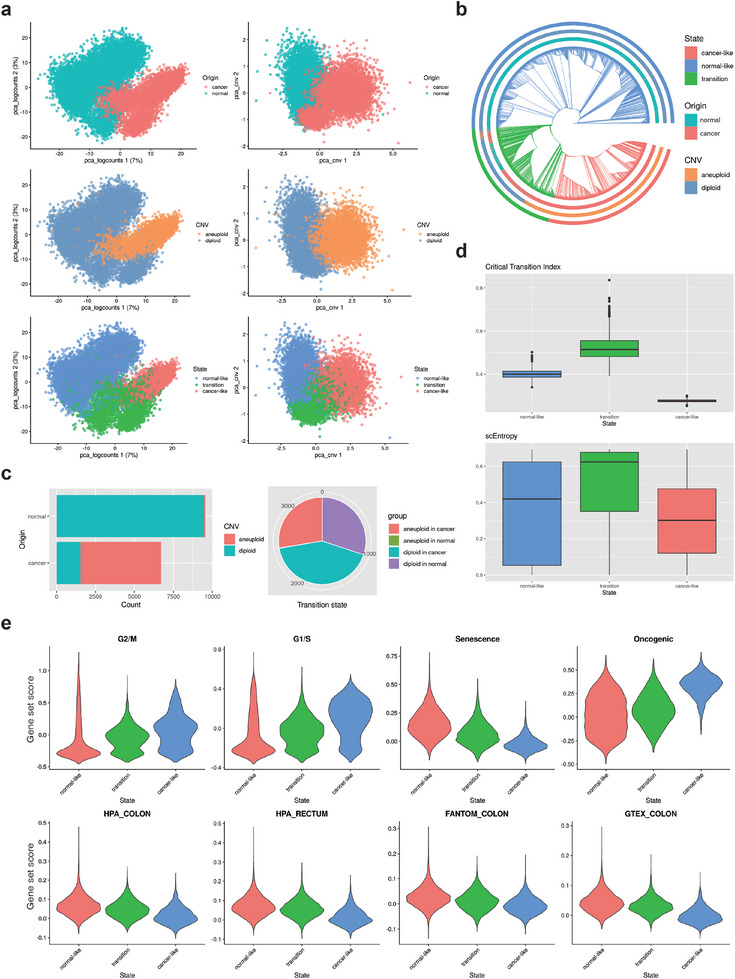
Identification of tumor transition state. a) Principal component analysis of scRNA‐seq data of cancer and adjacent normal tissues in transcriptomic (left column) or CNV (right column) spaces. Cells are colored by their originated tissues (top), their aneuploidy (middle), and their cell state (bottom). b) Phylogenetic tree reconstructed from single‐cell CNV profiles. It was inferred by the neighbor joining method and colored by cell origin, aneuploidy, and predicted state. c) Cell composition of normal and cancer tissues (left) and the transition state (right) in terms of aneuploidy. d) Critical transition index and single‐cell entropy (scEntropy) for normal‐like, transition, and cancer‐like states. e) Gene set scoring of cancer‐related signatures (top) and normal colorectal tissue signatures (bottom).

To develop REVERT, we produced single‐cell RNA sequencing (scRNA‐seq) data from patient‐derived organoid samples taken from both cancer and adjacent normal tissues of a colorectal cancer (CRC) patient (**Figure** **2**a, top). To order cells along with the accumulation of genetic alterations, we utilized a copy number variation (CNV) inference algorithm, CopyKAT,^[^ ^19^ ^]^ to predict aneuploidy at single‐cell level from scRNA‐seq data (Figure 2a, middle). From this, we can distinguish between cancerous and normal cells, and also reveal an intermediate clone comprising a mixture of various cellular states including diploid cells of cancer origin, aneuploid cells of normal origin, etc. The single‐cell copy numbers were then used to reconstruct a phylogenetic tree which describes the sub‐clonal relationships among cells and summarizes their chronicle of tumorigenesis (Figure 2b). We found that some clades are characterized by heterogeneous cell populations which include normal and cancer‐originated cells or diploid and aneuploid cells, in contrast to the homogeneous cell populations found at both the beginning and end of the tree. Cells in these heterogenous clades are in an intermediate state between normal and cancer cell states (Figure 2a, bottom), which we define as a tumor transition state (Figure 2b,c, and Figure S1, Supporting Information, see the Experimental Section). This heterogeneity in the transition state is relevant to the instability at a tipping point and can be characterized by the critical transition index^[^ ^1a^ ^]^ which is defined as the ratio of the average of gene‐gene correlation to the average of cell–cell correlation, and the single‐cell transition entropy (scEntropy)^[^ ^22^ ^]^ which measures cell plasticity. Both quantities were found to be significantly higher at the transition state than that at the normal‐like state or cancer‐like state, indicating that cells in the transition state are under an unstable condition at the tipping point and are prone to transitioning toward specific stable states (Figure 2d). Furthermore, we found that cells in the transition state exhibit intermediate gene set scoring of cancer‐related signatures, such as cell cycle, senescence, and oncogenesis, as well as the signatures of normal colon and rectum tissue (Figure 2e). In summary, cells in the transition state share similar genetic backgrounds (Figure 2b) but display heterogeneous transcriptional profiles, reflecting diverse phenotypic behaviors. In the following section, we present a dynamic network model for the transition state, capturing the dynamics of gene expression changes through a representative gene regulatory network common to cells within this state.

### 2.3 Reconstructing a Mechanistic GRN of the Transition State

**Figure 3:**
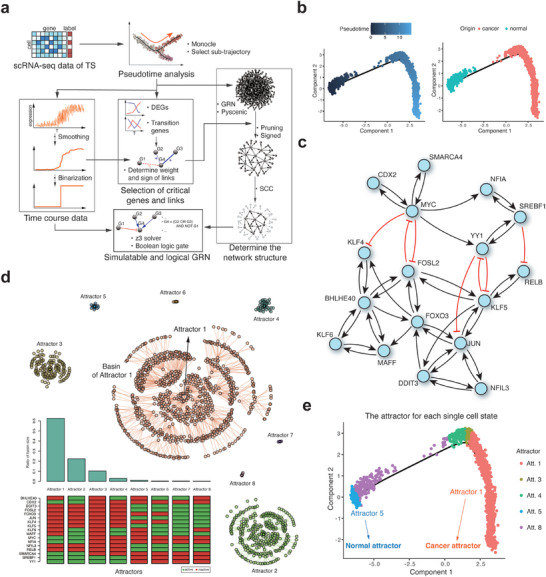
Reconstruction of dynamic network model for the transition state of colorectal cancer. a) Schematic overview of reconstructing a dynamic GRN model for the tumor transition state. Binarized single‐cell gene expression data and prior knowledge of the GRN structure are integrated to infer Boolean logic rules. b) Pseudotime trajectory of the tumor transition state inferred by Monocle. A specific trajectory was selected as the path from normal to cancer origin cells. c) Network structure of strongly connected components (SCCs) extracted from the GRN. Red links indicate inhibitory regulations, whereas black links represent activating regulations. d) State transition diagram and the molecular profiles of the attractors in the inferred Boolean network model. Attractors were sorted according to their basin size. We considered only 1000 random initial states and obtained a total of nine attractors, including eight point attractors and one cyclic attractor. The cyclic attractor is not shown in the figure as its corresponding basin size is the smallest. e. Attractors for each single‐cell state as an initial state. Cells in the earliest or latest pseudotime converge to Attractor 5 (normal attractor) and Attractor 1 (cancer attractor), respectively.

In the transition state, cells characterized by normal features can coexist with those exhibiting cancer features. These cells may undergo diverse trajectories characterized by stochastic transitions between the normal and cancer cell states. REVERT selects a specific trajectory, such as the path from the normal to cancer cell states to construct a dynamic network model based on Boolean logical regulatory rules using the time course data along with the path (**Figure** **3**a,b). To identify the optimal Boolean rules for a gene that fit well the selected time course data, we adopted a score function that quantifies the extent to which the predicted output of a Boolean function agrees with the observed pseudotime output, which was implemented in Pseudotime‐network‐inference^[^ ^12^ ^]^ using the Z3 solver. The construction of simulatable logical GRNs was achieved by integrating binarized gene expression data and the structural information of an initial GRN (Figure 3a).

Along the pseudotime trajectory, we curated genes of interest, including both differentially expressed genes (DEGs) and transition genes that are expected to be critical in tumorigenesis (Figure S2, Supporting Information). The direct binarization of scRNA‐seq data frequently results in significant temporal fluctuations, attributed to the inherent stochastic nature of single‐cell data. These fluctuations may not comprehensively capture the overall characteristics of the entire trajectory. Therefore, a smoothing process preceding the binarization procedure was undertaken using a moving window approach, yielding a moving‐averaged expression profile across pseudotime (Figure S3, Supporting Information). The smoothed gene expression data were then used to refine the initial backbone of the GRN by filtering highly correlated genes and assigning causal relationships and regulatory signs to their interactions. Considering the pivotal roles of feedback loops in shaping the dynamic behaviors of the entire network, we proceeded to extract strongly connected components (SCCs), wherein every node is reachable from every other node (Figure 3c). The resulting subnetwork was used for inferring Boolean regulation logical rules for each node based on the binarized expression data (Figure S4, Supporting Information) (see the Experimental Section for details). To determine the best rules for each gene regulation, we defined the score function for input‐output pairs from the pseudotime order (Figure S3, Supporting Information). For genes where multiple rules with the highest scores were derived, all the rules were combined together using the logical OR operation (Tables S1 and S2, Supporting Information).

Once the Boolean regulation rules of the network were established, Boolean simulations can predict the configuration of attractors, the states to which the Boolean network eventually converges depending on initial states, and their corresponding basins, the collection of all network states converging to the same attractor, which forms a state transition diagram showing the attractor landscape (Figure 3d). Attractor analysis revealed that the four largest basins (Attractor 1 to 4) exhibit more similar patterns of gene expression of the corresponding attractors than the remaining basins, and cover most of the state space. To ascertain the specific cellular phenotypes corresponding to each attractor, we analyzed the attractor state of individual single cells, using their binarized gene expression values as initial states (Figure S5, Supporting Information). The results show that cancer cells in the latter part of pseudotime converge to the Attractor 1, whereas normal cells at the earliest pseudotime remain at the Attractor 5 (Figure 3e), indicating that Attractors 1 and 5 correspond to the cancer cell and normal cell attractors, respectively. The cancer attractor delineates the specific molecular profiles that underlie cancer‐related phenotypes. In particular, MYC, a well‐known oncogene associated with tumor initiation and progression in cancer,^[^ ^23^ ^]^ is upregulated in the cancer attractor. Moreover, genes elevated at the cancer attractor, such as CDX2 (Caudal Type Homeobox 2),^[^ ^24^ ^]^ YY1 (Yin Yang 1),^[^ ^25^ ^]^ SREBF1 (Sterol Regulatory Element‐Binding Transcription Factor 1),^[^ ^26^ ^]^ and SMARCA4 (SWI/SNF Related, Matrix Associated, Actin Dependent Regulator of Chromatin, Subfamily A, Member 4),^[^ ^27^ ^]^ were implicated in promoting CRC progression by regulating processes such as cell proliferation, invasion, and metastasis, although their roles may vary depending on the context of CRC. Conversely, the normal attractor exhibits the gene expression patterns opposite to those of the cancer attractor (Figure 3d).

Together, these results show that REVERT can construct a dynamic network model for the tumor transition state based on scRNA‐seq data and effectively simulate Boolean regulation functions to elucidate the attractor landscape governing the transition between normal and cancer cell states. We may expect that two major attractors corresponding to normal and cancer attractors would manifest in the transition state (see Figure 1a), unlike our case where the basin size of the cancer attractor markedly exceeds that of normal attractor (Figure 3d). This may be attributed to our initial selection process where we specifically chose a trajectory from normal cells to cancer cells. Consequently, the inference of Boolean regulation functions based on such a trajectory is inherently biased to the selected dynamics which eventually leads to cancer cell states. Indeed, there may exist many alternative trajectories, including a trajectory from cancer cells to normal cells, owing to the stochastic nature of the transition state. The ultimate composition of the attractor landscape depends on the chosen initial trajectory. We note that the presence of the normal attractor, even though its basin size is considerably smaller than that of the cancer attractor, may open up the possibility for cancer reversion.

### 2.4 Quantifying the Attractor Landscape to Identify Optimal Therapeutic Targets for Cancer Reversion

**Figure 4:**
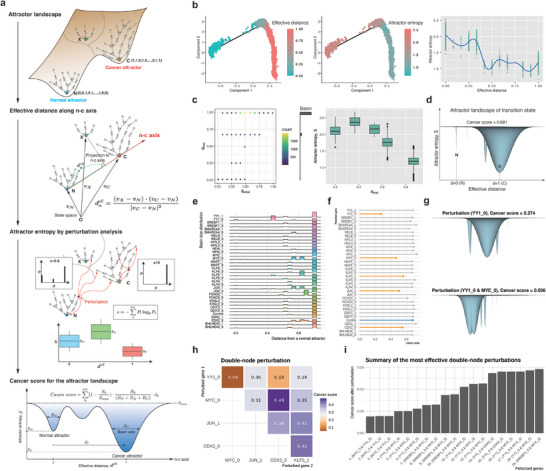
Quantification of the attractor landscape to identify optimal therapeutic targets for cancer reversion. a) Workflow for quantifying the attractor landscape to obtain the cancer score. The discrete attractor landscape is transformed into continuous landscape in n‐c axis by introducing effective distance (projection onto the n‐c axis), attractor entropy (depth of valleys), and basin size (width of valleys) for each attractor. The cancer score quantifies the malignancy of the landscape by calculating the volume of valleys, scaled by their proximity to the cancer attractor. b) Mapping of single cells from the transition state onto the attractor landscape, showing effective distance (left) and attractor entropy (center) along pseudotime. Scattering cells onto an effective distance‐attractor entropy plane enables a rough visualization of the landscape (right). c) Basin size (left) and attractor entropy (right) of attractors for 10000 random initial states. The effective distances of five attractors are represented on the y‐axis, and the effective distances of initial states are on the x‐axis. The histogram on the y‐axis indicates the basin size of the five attractors. d) 2D representation of the attractor landscape for the transition state. The relative positions, depths and widths of the valleys were determined by the effective distance, attractor entropy, and basin size obtained in (c). N and C represent the normal cell and cancer cell attractors, respectively. e,f) In silico perturbation analysis of gene expression, showing changes in basin size (e) and cancer score (f) in response to gene knockout or overexpression. The cancer score of the control without any perturbation in colored in blue, whereas the five most effective cases are colored in orange. “0” and “1” indicate knockout and overexpression of the corresponding gene, respectively. g) Changes in the attractor landscape for the knockout of YY1 (top) and the double knockout of YY1 and MYC (bottom). h) Cancer scores for double knockout among the top five effective perturbations in (f). i) Summary of the most effective double‐node perturbations for various hyperparameter sets.

The attractor landscape of a Boolean network serves as a discrete representation of potential energy landscape of networks states, offering comprehensive insights into the relationship between binarized network states, namely separated attractors and their corresponding basins upon a state transition diagram (Figure 3d). Nevertheless, the discrete nature of this landscape imposes limitations in assessing the robustness of attractors to external perturbations and discerning their relative stability (corresponding to depths of attractors) in the landscape, which are crucial for understanding system resilience and systematically exploring potential drug targets for cancer reversion. To estimate the relative depths of attractors and ultimately quantify the attractor landscape of a Boolean network in the context of malignancy, we postulated the presence of a virtual axis extending from the normal attractor state to the cancer attractor state in the state space (**Figure** **4**a). The gene expression vector of any given state can be projected onto this axis, enabling the calculation of the Euclidean distance from the normal attractor along the normal‐cancer (n‐c) axis, termed the effective distance, denoted as *d^eff^* (see the Experimental Section). An effective distance near 0 or 1 indicates that the given state is close to a normal or cancer attractor, respectively.

While the effective distance serves as an indicator of the relative positioning of attractors within the landscape, perturbation analysis offers a method to evaluate their depth by observing the uncertainty of perturbed steady states in response to random perturbations, which we denote as “attractor entropy” (see the Experimental Section and Figure 4a). A high attractor entropy of a state indicates a high probability of converging to other attractors, suggesting that this state may belong to a shallow energy well in the attractor landscape. We define the averaged attractor entropy over all states within a basin of attraction as the depth of the corresponding potential energy well, representing the vertical position of the attractor.

The continuous attractor landscape can be synthetically constructed by utilizing the effective distance, the averaged attractor entropy, and the basin size of each attractor. These metrics respectively determine the position along the n‐c axis, the vertical depth, and the width of the corresponding potential energy wells. Such a virtual landscape holds the capability to provide insights into the malignancy of a given cell state. For instance, a landscape wherein the cancer attractor represents a significantly deeper and wider valley compared to the normal attractor implies a highly malignant cell state. To quantify such malignancy of a given attractor landscape, we introduced a cancer score. This score can be conceptualized as the volume of water filling the valleys, scaled by the proximity of individual valleys to the cancer attractor. Specifically, this is calculated as the product of the area of each attractor valley and the effective distance of each attractor along the n‐c axis (see the Experimental Section and Figure 4a).

We used the aforementioned metrics to track cellular trajectories over time by mapping single cells in the transition state onto the landscape (Figure 4b). Cells at earlier pseudotime are closer to the normal attractor, whereas those at later time are positioned near the cancer attractor. A reduction of entropy was observed among cells at later pseudotime, indicative of deeper energy wells within the landscape, suggesting that cells at later pseudotime occupy more stable states. We further examined the attractors for random initial states, along with their associated metrics to elucidate entire potential energy landscape of the transition state. Our results revealed that the five attractors are projected onto the n‐c axis, with the cancer attractor at *d^eff^* =  1 forming a significantly larger and deeper valley compared to the normal attractor (Figure 4c,d). Intriguingly, the normal attractor, despite having a small basin, exhibits a relatively deep valley, suggesting that the normal attractor forms a distinct valley characterized by high robustness to external perturbations. The coexistence of distinct attractors, including both normal and cancer attractors, represents a hallmark feature of the transition state. Our analysis also revealed that the cancer score for the transition state is high, attributed to the excessively large valley of the cancer attractor (Figure 4d).

The significance of attractor landscape and its quantification becomes evident when exploring potential therapeutic drug targets. By systematically perturbing the GRN in silico, such as altering connections, adjusting node states, or introducing external inputs, we can effectively control the dynamics of cellular systems. Such in silico perturbation enables us to reshape the landscape, thereby facilitating control over the system toward a desired cell state like a normal state as shown in this case study. To identify the optimal drug targets for cancer reversion, we conducted in silico perturbation analysis by perturbing gene expression, either by fixing it to 0 (knockdown) or 1 (overexpression). REVERT can simulate changes in the attractor landscape in response to gene perturbations, including basin size, effective distance, and attractor entropy of attractors (Figure 4e and Figure S6, Supporting Information). It then provides a list of effective target genes based on the resulting cancer scores (Figure 4f). Among the identified target genes, the knockdown of YY1 emerged as the most effective target gene, leading to the creation of a new attractor closer to the normal attractor with a significantly large basin size (Figure 4e). Moreover, the basin size and attractor entropy of the normal attractor increased and decreased, respectively, compared to those of the cancer attractor (Figure S6, Supporting Information, and Figure 4g). These results suggest that inhibiting YY1 might be effective as an anti‐cancer strategy. To identify the drug targets for cancer reversion beyond anti‐cancer therapy, we conducted a double‐node perturbation analysis (Figure S7, Supporting Information). The double knockdown of YY1 and MYC resulted in a significant decrease in the cancer score to 0.056, corresponding to the disappearance of the cancer attractor and the emergence of dominant normal and near‐normal attractors in the landscape (Figure 4g,h and Figure S8, Supporting Information). The double knockdown of YY1 and CDX2 was the second most effective strategy, whereas targeting MYC and CDX2 resulted in a less favorable outcome compared to targeting MYC alone (Figure S9, Supporting Information). This indicates that the combination of YY1 and MYC might be the most synergistic in promoting cancer reversion.

REVERT relies on multiple hyper‐parameters, including the number of DEGs along pseudotime, the width of smoothing windows, and the time step size for input‐output pairs in calculating the scoring function. These hyper‐parameters can slightly influence the resulting network and, consequently, impact the identification of optimal drug target candidates for cancer reversion. To ensure the robustness of our results, we constrained these hyper‐parameters. Specifically, we limited the network size to 10∼30 nodes to avoid highly complex networks with an excessive number of attractors. In addition, we set a threshold for the initial cancer score of the transition state to be exceeding 0.5 to exclude trivial cases for cancer reversion. We then collected the most effective target genes showing cancer scores less than 0.15 after perturbations among the cases for various hyper‐parameter sets. Of note, the double knockdown of YY1 and MYC consistently showed low cancer scores after perturbations in the majority of cases (Figure 4i and Table S3, Supporting Information), confirming that the combination of YY1 and MYC may exhibit synergistic effects, rendering them promising target genes for cancer reversion.

### 2.5 Identification of the Optimal Target Gene for Cancer Reversion

**Figure 5:**
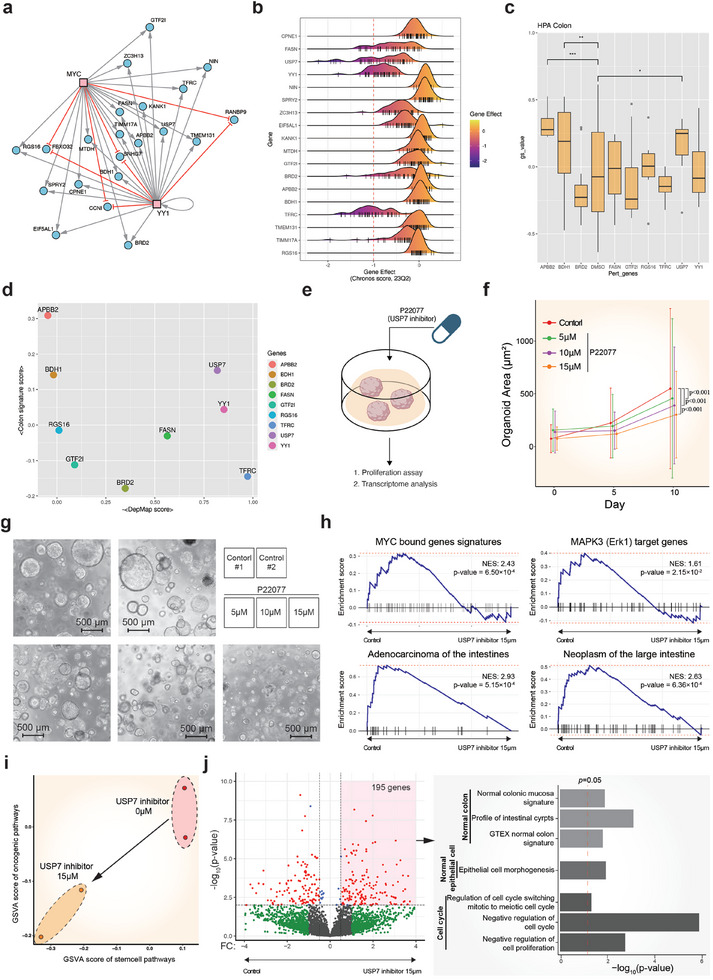
Experimental validation of the optimal target gene for cancer reversion. a) Common target genes positively (gray lines) or negatively (red lines) regulated by both MYC and YY1 transcription factors. b) Cancer dependency scores of positively regulated common target genes derived from CRISPR knockout screen datasets (DepMap) across various colon cancer cell lines. Negative scores indicate cell growth inhibition upon gene knockout. Bars in the x‐axis represent given cell lines. c) Enrichment analysis of gene expression changes following genetic perturbation of colon cancer cell lines (HT29, LOVO, SW480, SW620, and HCT116) using the LINCS L1000 database. The normal colon gene signature was obtained from the Human Protein Atlas. d) Identification of USP7 as the optimal target gene for cancer reversion, based on combined scores for cancer dependency and normal signature enrichment. e) Schematic representation of the experimental methodology using the USP7 inhibitor P22077 to validate USP7 as a potential target for cancer reversion in colon cancer organoids. f) Quantification of the colon cancer organoids growth changes upon USP7 knockdown, depicting the relative growth rates at day 0, 5, and 10 post‐knockdown for varying concentrations of the USP7 inhibitor (0 × 10^−6^, 5 × 10^−6^, 10 × 10^−6^, and 15 × 10^−6^ m). The p‐value was calculated using repeated‐measures (RM) analysis of variance (ANOVA): *p* < 0.001. g) Representative images depicting the morphological changes and reduced growth of colon cancer organoids upon USP7 knockdown using the inhibitor P22077, compared to untreated control organoids. h) Gene set enrichment analysis (GSEA) results illustrating the significant downregulation of gene signatures associated with tumor formation upon USP7 knockdown in colon cancer organoids. i) Dot plot representation depicting the GSVA scores for stem cell pathways (x‐axis) versus oncogenic pathways (*y*‐axis) in USP7 knockdown organoids (orange dots) and control organoids (red dots). j) (Left) Volcano plot illustrating the differentially expressed genes between control organoids and USP7 knockdown organoids, with genes exhibiting significant upregulation upon USP7 inhibition highlighted in red. (Right) Bar plot depicting the results of GO analysis for the 195 positively differentially expressed genes.

MYC is a well‐known oncogene in CRC, with its upregulation being linked to tumorigenesis progression.^[^ ^23a^ ^]^ YY1 is also overexpressed in multiple cancer types, including CRC, where its overexpression is correlated with poor clinical outcomes.^[^ ^28^ ^]^ Therefore, therapeutic strategies that target these two key transcription factors could potentially enhance treatment outcomes for CRC patients. Nonetheless, there are significant limitations in using them as therapeutic targets. YY1 regulates the expression of numerous genes, increasing the risk of unintended consequences on normal cellular processes, including off‐target effects^[^ ^29^ ^]^ and cytokine release syndrome.^[^ ^30^ ^]^ MYC remains an undruggable therapeutic target due to the absence of well‐defined active sites on its protein structure where small molecules can bind to.^[^ ^31^ ^]^ Furthermore, its complete inhibition could affect normal homeostasis, as MYC plays a crucial role in various physiological processes associated with tissue generation.^[^ ^32^ ^]^ To overcome these limitations, we further investigated to identify alternative target genes regulated by both transcription factors. To identify such common target genes, we used the initially constructed backbone of the GRN obtained from the SCENIC algorithm. This network includes subsets of target genes that are either positively or negatively regulated by both MYC and YY1 (**Figure** **5**a). We first analyzed the cancer dependency of positively regulated common target genes using CRISPR screen datasets from the Cancer Dependency Map (DepMap). Most of these target genes exhibit gene effect scores less than 0 across various colon cancer cell lines, suggesting that their inhibition could potentially suppress the proliferation of the corresponding cell lines (Figure 5b). Notably, several genes, including USP7 and TFRC, have scores lower than ‐1, indicating their critical role in these cell lines. Further analysis was conducted by assessing the changes in enrichment scores for normal colon signatures in colon cancer cell lines following genetic perturbations, using data from the LINCS L1000 database.^[^ ^20^ ^]^ This revealed that the inhibition of several genes, such as APBB2, BDH1, and USP7, substantially enhances the normal colon signature within cancer cell lines (Figure 5c). By combining the scores for cancer dependency and normal signature enrichment, we identified USP7 as the optimal target gene for cancer reversion (Figure 5d).

### 2.6 Experimental Validation of the Predicted Cancer Reversion Target

USP7 (ubiquitin‐specific protease 7) plays important roles in cancer progression by deubiquitinating and stabilizing various oncogenic proteins like MDM2, FOXP3, and PTEN, thereby emerged as a promising therapeutic target.^[^ ^33^ ^]^ In colon cancers harboring APC mutations, USP7 acts also as a tumor‐specific Wnt activator by promoting the deubiquitination and stabilization of β‐catenin.^[^ ^34^ ^]^ Deletion of USP7 in colon cancers inhibits Wnt activation by restoring β‐catenin ubiquitination, facilitating differentiation and suppressing tumor growth. Furthermore, there is evidence that USP7 inhibition can selectively target acute myeloid leukemia (AML) cells while having minimal effects on normal cells.^[^ ^35^ ^]^ This selectivity is likely due to the specific roles of USP7 in regulating pathways and proteins that are aberrantly activated in cancer cells but not in normal cells. These findings make USP7 an attractive therapeutic target for cancer reversion in colon cancer cells.

To investigate the efficacy of USP7 as a potential target for cancer reversion in colon cancer organoids, we conducted USP7 knockdown experiments using the inhibitor P22077 in colon cancer organoids previously used in the process of network reconstruction. We subsequently evaluated the inhibition of cancer cell proliferation and the induction of the transcriptomic signature associated with normal colon cells (Figure 5e). From these experiments, we aimed to determine whether the inhibition of USP7 can lead to the reversion of colorectal cancer organoids. Of note, USP7 knockdown in colon cancer organoids significantly reduced the growth rate of the organoids (Figure 5f,g). Furthermore, gene set enrichment analysis (GSEA) showed that USP7 knockdown is significantly associated with the inhibition of tumor formation by downregulating the genes related to MYC and MAPK3 pathways (Figure 5h). These alterations in the transcriptome landscape were accompanied by the downregulation of genes associated with stem cell pathways (Figure 5i). These results suggest that USP7 exhibits anti‐cancer properties in colon cancer, which is consistent with our DepMap analysis result (Figure 5b) and previous experimental findings.^[^ ^33^ ^]^ Next, Gene Ontology (GO) analysis of positive DEGs upon USP7 knockdown revealed its significant association with the pathways implicated in normal colon epithelial cells and the genes related to the negative regulation of cell cycle and cell proliferation (Figure 5j), consistent with the previous L1000 analysis results (Figure 5c). Together, these findings indicate that knockdown of USP7 in colon cancer organoids can facilitate the reversion of them to more normal colon states.

The experimental validation of USP7 as a potential therapeutic target for cancer reversion, identified through REVERT, provides compelling evidence for the efficacy of dynamic network modeling of tumor transition states. The observed reversion of colon cancer organoids to a more normal state upon USP7 inhibition supports the predictions made by our mechanistic Boolean network models and attractor landscape analysis. These findings underscore the usefulness of our integrative framework, REVERT, which combines single‐cell transcriptomic data, dynamic network modeling, and in silico perturbation simulations, to identify and validate optimal therapeutic targets for cancer reversion.

## 3 Discussion

It has been an important challenge to develop a systems framework for controlling cell states based on omics data in the fields of diverse biological studies, drug discovery, stem cell engineering, and regenerative medicine. The key innovation of this study lies in the identification of potential targets for cancer reversion through the development of a dynamic network model focusing on the transition state in tumorigenesis. As described in Introduction, unlike developmental or differentiation processes, cancer progression involves structural changes in the GRN due to accumulation of mutations, making it difficult to capture the entire cancer progression dynamics through a single GRN framework. To overcome such difficulty, we solely focused on the transition state of cancer progression and constructed a GRN based on the single cells across the transition state. Both normal and tumor cells in this state may still share similar genetic alterations since they are located in the same or adjacent clades on the phylogenetic tree based on CNV characteristics. This similarity enables us to construct a single unified network model. By analyzing such a unified network model on the transition state, we were able to represent the attractors of both normal and tumor cells within a single attractor landscape, facilitating the identification of target genes that could potentially drive cancer reversion. Here, we presented REVERT, a computational framework for cancer reversion, which integrates dynamic network modeling and attractor landscape analysis using single cell transcriptomic data of paired organoid models derived from a colorectal cancer patient. REVERT can identify a key target gene as a reversion switch which may induce a transition from cancer cell states to normal cell states by controlling the attractor landscape of tumor transition states. Therefore, REVERT provides an opportunity for developing targeted therapeutic strategies for cancer reversion and advancing our understanding for cellular reprogramming processes.

CNVs, as permanent genomic alterations, are inherently irreversible. Our focus is, however, on functional and phenotypic reversibility rather than direct reversal of the CNVs themselves. In this study, the transition state is defined based on CNV‐driven network alterations. While CNVs establish the structural framework of the gene regulatory network, the state of the system within this framework can still be modulated. By targeting key regulatory genes within the dynamic network model that shape the attractor landscape, we propose strategies to reshape the attractor landscape of the transition state to effectively mimic that of the normal state. This approach enables the system to move toward a phenotype resembling the normal state, even under the constraints imposed by the CNVs. In essence, our model explores the potential for phenotypic plasticity within the constraints of irreversible genomic changes, offering insights into a new potential therapeutic strategy in cancer treatment.

MYC, one of the cancer reversion targets identified in our study, is a well‐established key regulator in cell fate determination. Our finding regarding MYC is partially attributable to the fact that the gene regulatory network we constructed is informed by prior knowledge of TF‐TG regulatory networks. Nevertheless, this finding underscores that REVERT is reliable and capable of accurately identifying critical components of gene regulatory networks. Although MYC was predicted as a cancer reversion target, its single inhibition does not significantly alter the size or width of the cancer attractor and, consequently, does not substantially reduce the cancer score (Figure S9b, middle, Supporting Information). Interestingly, while MYC inhibition causes only a minimal increase in the width of the normal attractor, it unexpectedly results in a substantial increase in the depth of the normal attractor. These changes in the attractor landscape suggest that while MYC contributes to the stabilization of the normal attractor, thereby opening a possibility for cancer reversion, it has limited effectiveness in achieving cancer reversion on its own. This emphasizes the need for additional co‐targets, such as YY1, to facilitate more effective cancer reversion.

There have been accumulated evidences showing that stable cellular states in Waddington's epigenetic landscape can be represented by the underlying core molecular regulatory circuits.^[^ ^36^ ^]^ These circuits often have the form of double‐negative feedback loops, referred to as bistable toggle switches, which play a crucial role in driving cell state transitions.^[^ ^36^, ^37^ ^]^ Cross‐repression between transcription factors (TFs) in such circuits is instrumental in maintaining the stability of cellular states and facilitating transitions between them. Our analysis revealed MYC and YY1 as key elements of double‐negative feedback loops which interconnect core regulatory circuits associated with normal and cancer attractors (Figure 3c), indicating that these TFs can potentially act as master regulators for cancer reversion. REVERT offers a comprehensive approach for systematically identifying core regulatory networks and their master regulator across various cell‐fate changes. By combining the principles of bistable toggle switches and core regulatory circuits, REVERT provides a useful tool for elucidating the mechanism underlying cell state transitions and identifying key targets for cellular reprogramming not only in cancer but also across various cell fate changes.

The mechanistic modeling of REVERT can be characterized by several key features that distinguish it from existing methods. First, REVERT employs Boolean network modeling to capture the essential dynamic behaviors of cells on the basis of prior knowledge of the molecular regulatory network structure. The landscape control approach, grounded in continuous dynamical systems, models GRNs using differential equations and provides detailed insights into the energy landscape of a system.^[^ ^38^ ^]^ This methodology excels in quantifying changes in energy landscapes, such as basin depths, barrier heights, and transition probabilities, offering a rich representation of the stability and dynamical behavior of cell states. It is particularly advantageous for understanding complex attractor basins and quantifying the likelihood of transitions between cell states under different perturbations. Furthermore, it facilitates the identification of control strategies by pinpointing key regulators that drive transitions, offering direct insights into potential therapeutic targets. While representing gene expression levels by binarized activity states may result in the loss of details on the cellular behavior, Boolean network modeling still have several advantages: it does not require kinetic parameters to be estimated, facilitates interpretability, provides a simple dynamic approach to model GRNs, and enables straightforward simulation analysis for network perturbations. Recently, single‐cell transcriptomic data were employed to construct Boolean network models since Boolean models have the advantage of being robust to data uncertainty including stochastic variations inherent in single‐cell data. However, existing approaches either rely on simple correlation methods for network inference without considering prior knowledge (e.g., BTR^[^ ^13^ ^]^ and Pseudotime‐network‐inference^[^ ^12^ ^]^), or cannot handle direct feedback loop circuits between two genes that are actually ubiquitous in gene interaction networks (e.g., IQCELL^[^ ^15^ ^]^). REVERT overcomes these limitations by integrating prior knowledge and single cell transcriptome data, resulting in improved accuracy and robustness in developing Boolean network models.

Second, the attractor landscape analysis of REVERT enables us to identify target genes that can induce the transition from a cancer cell state to a desired normal cell state. This approach also holds significant potential for not only elucidating the mechanisms underlying cancer initiation but also investigating other various biological phenomena on cell fate changes such as in developmental processes or cellular differentiation. For these applications, the initial cellular state and the desired target state must be clearly defined and characterized in the high‐dimensional cellular state space based on transcriptome. In addition, to enhance robustness to the choice of hyperparameters in the attractor landscape analysis, ensemble models can be considered for identification and prioritization of control targets.

Third, the quantification of the attractor landscape in REVERT enables systematic in silico gene perturbations. The attractor landscape of a Boolean network model contains multidimensional information and therefore it is difficult to directly compare the relative stability of attractors. To resolve this, REVERT projects attractors onto the n‐c axis and employs the concept of attractor entropy to quantify the malignancy of a given attractor landscape in terms of a cancer score. In this way, REVERT enables the identification of optimal targets through single or double gene perturbation simulations. We note, however, that the projection process onto the n‐c axis inevitably leads to information loss. In addition, for tumorigenesis that bifurcates into two distinct subpopulations, REVERT focuses only on the primary tumorigenic trajectory. Hence, there is a need to develop methods that can allow higher dimensional analysis, enabling a more comprehensive understanding of the attractor landscape.

In this study, to simplify the scope of the transition state as much as possible, we defined the tumor transition state using normal and tumor cells that exist along a single lineage. Therefore, the statement that cells in the tumor transition state share the same mutational background reflects the specific context of our analysis, where the single‐cell transcriptomic data were derived from tumor and matched normal organoid models, representing a closely related population of cells within the tumor. This allowed a focused exploration of the dynamic changes occurring in cells likely originating from a common mutational lineage. In broader biological contexts, it is possible for transition cell states to arise from different genetic backgrounds. For instance, in heterogeneous tumor microenvironments, subclones with distinct mutational profiles could exhibit similar transitional behaviors due to shared regulatory pathways or convergent phenotypic pressures. While our current analysis focused on a homogeneous mutational context to simplify the network inference, future work should explore how diverse genetic backgrounds might influence the transition state.

The single‐cell data used for network construction and pseudotime inference were derived from a tumor, and therefore, the “normal” and “cancer” cell states in our analysis represent tumor‐associated normal‐like and cancer‐like states rather than true healthy or cancerous tissue. The pseudotime trajectory was computationally inferred, with its directionality determined based on transcriptional similarities and differences. To approximate a transition from normal‐like to cancer‐like states, we defined the starting point of the pseudotime as cells with transcriptional profiles most similar to normal‐like cells and the endpoint as cells aligning with cancer‐like profiles. This interpretation relies on the ergodic hypothesis (as illustrated in Figure 1), which posits that a snapshot of a cell population at a single sampling time point can represent the full spectrum of intermediate cellular states along the normal‐to‐tumor transition trajectory. This approach has shown a potential in modeling lineage commitment in the hematopoietic system.^[^ ^39^ ^]^ Similarly, the diversity of cellular states observed within the tumor at a given time point can reflect different stages of progression from normal‐like to cancer‐like states.

There is a growing interest in pseudotime analysis of single‐cell transcriptomic data to investigate dynamic gene expression programs over cell fate changes such as differentiation, trans‐differentiation, and reprogramming. However, in the context of tumorigenesis which involves the accumulation of genetic alterations, it is crucial to incorporate genomic information for accurate inference of pseudotime trajectories and identification of tumor transition states. In this study, we incorporated CNVs inferred from scRNA‐seq data to identify tumor transition states. However, it is needed to consider more accurate genetic alterations instead of relying on the inferred data. One possible approach is to adopt multiomics single cell sequencing data which can enable to jointly analyze both genomic and transcriptomic information from the same cell, although it is still challenging to simultaneously measure the very small quantity of DNA and RNA present in a single cell. Alternatively, single‐cell long‐read sequencing data^[^ ^40^ ^]^ can be utilized to detect single nucleotide variants (SNVs) in exonic regions, providing a promising way to identify tumor transition states with higher resolution. By integrating genomic and transcriptomic data through these emerging single‐cell multiomics techniques, we can seek for a more comprehensive understanding of the molecular mechanisms underlying tumor transition states and thereby developing ultimate targeted therapies for cancer reversion.

## 4 Experimental Section

### Establishment of Patient‐Derived Paired Normal Colon and Colon Cancer Organoids

Tissues from human colons were obtained from the Seoul National University Hospital (Seoul, Korea). After removal, each tumor and its matched normal tissue was used to prepare for cultures. This study was approved by Seoul National University Hospital (approval number: 1710‐102‐896). Tissues were chopped and minced using a gentleMACS Dissociator (Miltenyi Biotec, Bergisch Gladbach, Germany). Dissociated samples were passed through a 70 µm cell strainer to remove large tissue fragments. Isolated tumor cells were embedded in Matrigel and seeded in 24‐well plates, followed by the addition of organoid media. Organoid medium was refreshed thrice weekly. The organoid medium consisted of 50% L‐WRN conditioned media,^[^ ^41^ ^]^ 50 ng mL^−1^ recombinant human EGF (PeproTech, USA), B27 supplement (Invitrogen, USA), 1.25 × 10^−6^ m N‐acetyl cysteine (Sigma‐Aldrich, USA), nicotinamide (Sigma‐Aldrich, USA), 3 × 10^−6^ m SB202190 (Sigma‐Aldrich, USA), 500 × 10^−9^ m A83‐01 (Tocris, UK), 10 × 10^−9^ m prostaglandin E2 (Sigma‐Aldrich, USA), 100 µg mL^−1^ Primocin (InvivoGen, USA), and 1% antibiotics (10 000 units mL^−1^ penicillin, and 10 000 µg mL^−1^ streptomycin; WELGENE, Korea) in advanced DMEM/F12 (Gibco, USA). L‐WRN, a secretion cell line of Wnt3A, R‐spondin1, and Noggin, was sourced from ATCC (USA).

### Establishing and Preprocessing Single‐Cell RNA Sequencing Libraries from Dissociated Organoids using 10x Genomics

Organoids were dissociated into single cells for RNA sequencing analysis. Following rinsing with phosphate‐buffered saline (PBS), organoids were enzymatically dissociated using TrypLE Express (Gibco) and incubated at 37 °C for 15 min. During incubation, the mixture was pipetted every 5 minutes to promote complete dissociation. The enzymatic reaction was halted by adding Dulbecco's Modified Eagle Medium (DMEM) supplemented with 10% fetal bovine serum (FBS). The resultant cell suspension was filtered through a 40 µm cell strainer to achieve a uniform single‐cell suspension. Cell viability and density were assessed using Trypan Blue exclusion with a hemocytometer to ensure optimal cell concentration for sequencing. For library preparation, the single‐cell RNA sequencing library was constructed using the Chromium Single Cell Instrument (10x Genomics), following the manufacturer's protocol. The process involved loading cells into a Chromium Chip B where cells, master mix, and partitioning oil were combined to generate single‐cell gel beads in emulsion (GEMs). Post GEM‐RT reaction, GEMs were broken, and the barcoded cDNA was isolated, cleaned using DynaBeads MyOne Silane Beads (Invitrogen), and amplified by PCR. The quality and quantity of the amplified cDNA libraries were evaluated using an Agilent 4200 Tapestation system (Agilent Technologies). The libraries were sequenced on an Illumina Nova‐seq 6000 system, employing paired‐end sequencing as recommended by the manufacturer. This method ensures the generation of high‐quality data for transcriptomic analysis. In addition, apoptotic cells that express more than 15% mitochondrial transcripts were excluded, as these are considered low‐quality cells. Following this filtering step, 18049 cells were retained for further analysis. Each cell underwent log normalization by scaling to a constant total read count per cell (100 000), followed by log transformation. For visualization, principal component analysis (PCA) was initially conducted using 2000 highly variable genes for dimensionality reduction. Subsequently, PCA dimensions were utilized to project into 2D space. All these analyses were conducted using the Seurat v4.0.2 package in R (v4.2.0).

### Identifying SCNA from Single‐Cell Transcriptome Data with CopyKAT

To identify somatic copy number alterations (SCNAs) from single‐cell transcriptome data, CopyKAT (version 1.0.3)^[^ ^19^ ^]^ with default settings was used. This analysis included both aneuploid and diploid cells from external validation biopsies, focusing on paired organoid single‐cell transcriptome data.

### Reconstruction of a Phylogenetic Tree from Inferred SCNAs in Single‐Cell Transcriptome Data

Our goal was to construct a phylogenetic tree based on SCNAs inferred from single‐cell transcriptome data. Using the bionj function in the ape package,^[^ ^42^ ^]^ a neighbor‐joining tree was created to estimate distances between cells. The resulting phylogenetic tree was visualized with the ggtree package, emphasizing cells in the transition state, where cancer and normal cells coexist during the transitional stage of tumorigenesis.

### Gene Set Scoring

The cancer‐related gene set including cell cycle, senescence, and oncogenesis is the used genes in previously defined meta‐programs of malignant cells.^[^ ^43^ ^]^ Normal colon gene signatures were obtained from various databases: the Human Protein Atlas (HPA),^[^ ^44^ ^]^ FANTOM,^[^ ^45^ ^]^ and the Genotype‐Tissue Expression project (GTEx).^[^ ^46^ ^]^ The gene sets obtained from these databases included genes that exhibit at least five‐fold higher level of expression in the colon compared to other tissues. Specifically, this resulted in 196 genes from HPA, 181 genes from FANTOM, and 480 genes from GTEx. Gene set scores were computed using the AddModuleScore function in Seurat.

### Defining Transition State by Identifying Heterogeneous Cell Populations in the Phylogenetic Tree

The transition state in tumorigenesis is defined as an intermediate state where cells exhibit mixed characteristics of normal and cancer cell states. To identify this state, a phylogenetic tree based on inferred CNVs was constructed, capturing the evolutionary relationships among cells. The tree was divided into 30 smaller clades to analyze the degree of heterogeneity within each clade (Figure S1a). Shannon entropy (*H*) was calculated for each clade to quantify the mixture of normal and cancer cell populations

(1) $$H=-\left(\frac{N_{n}}{N_{n}+N_{c}}\cdot \log_{2}\frac{N_{n}}{N_{n}+N_{c}}+\frac{N_{c}}{N_{n}+N_{c}}\cdot \log_{2}\frac{N_{c}}{N_{n}+N_{c}}\right)$$

where *N_n_* and *N_c_* is the number of normal and cancer cells in the clade, respectively.

Among the intermediate clades in the tree, the transition state was identified as the region where entropy showed a marked increase, indicating a significant blend of normal and tumorigenic characteristics (Figure S1b, Supporting Information).

### Critical Transition Index

Gene‐gene correlation reflects the co‐expression relationships between genes, while cell–cell correlation measures the similarity of gene expression profiles across individual cells, reflecting the extent to which cells within a population share similar transcriptional states. Critical transitions are often characterized by a loss of stability in the current attractor state, accompanied by increased variability and reduced coordination among system components. These shifts can lead to increased heterogeneity in cell–cell correlation while simultaneously altering gene–gene interactions. The ratio of these correlations reflects the interplay between these two dimensions, which can serve as a potential marker for the critical transition.

### Data Preparation and Pseudotime Inference of Transition Cells

First relevant genes were filtered in the transition state, such as highly variable genes (HVGs) and colon marker genes, and then conducted pseudotime analysis with those genes. While REVERT uses monocle2^[^ ^47^ ^]^ for pseudotime analysis, alternative tools like Slingshot^[^ ^48^ ^]^ can also be employed. In this study, monocle2 for pseudotime analysis was utilized to infer cellular trajectories. monocle2 allows for the generation of various trajectories depending on hyperparameter settings, resulting in different representations of cell state transitions. For instance, possible trajectories include normal origin cells progressing toward tumor origin cells, tumor origin cells transitioning back to normal origin cells, or normal origin cells bifurcating into two distinct tumor cell populations. Among these possibilities, the trajectory that progresses from normal origin cells to tumor origin cells was specifically selected. This selection was guided by its biological relevance, as it represents the transition from normal to tumor states, which is the main focus of our study. In addition, trajectories that align with known biological processes were carefully evaluated and selected to ensure the analysis remains meaningful and interpretable. Along this trajectory, genes were identified, including DEGs or switching genes by GeneSwitches,^[^ ^8a^ ^]^ that are expected to be critical along the trajectory. Our analysis only considered those cases where the final number of identified genes exceeds 1000.

### Generation of Binarized Gene Expression Data

To determine Boolean regulation rules for each gene, binarized time course data of gene expression as well as structural information of the gene regulatory network was needed. Binarizing gene expression values can lead to substantial temporal fluctuations due to the inherent stochastic characteristics of single cell data. Such fluctuations may not allow us to effectively capture the overall characteristics of the entire trajectory. Therefore, a smoothing process preceding the binarization procedure was performed using a moving window, resulting in a moving‐averaged expression profile across pseudotime. The window width was set to less than 10% of the total cell count.

### Determining the Structure of a GRN

REVERT exploits an initial backbone of the GRN as prior knowledge, from which a subnetwork is extracted according to the gene expression profiles associated with the trajectory. It began by performing pseudotime analysis to define a trajectory from normal cells to tumor cells. Using SCENIC (Single‐Cell Regulatory Network Inference and Clustering),^[^ ^49^ ^]^ a prior knowledge‐based GRN from the single‐cell transcriptomic data along this trajectory was constructed. SCENIC identifies transcription factor (TF)‐target gene (TG) relationships based on known TF binding motifs and cis‐regulatory elements, providing an initial regulon structure. SCENIC employs RcisTarget, a tool that uses motif discovery and enrichment analysis to identify transcription factor (TF)‐target gene (TG) regulatory relationships based on prior knowledge. Specifically, RcisTarget integrates databases of TF binding motifs and cis‐regulatory elements, mapping them to genes with enriched motifs in their regulatory regions. This approach enables the identification of putative TF‐TG interactions grounded in established biological knowledge. GRNs typically contain a large number of genes and links, which may render dynamic simulations computationally impractical. Moreover, some genes and links identified in the initial network may not contribute meaningfully to the dynamics observed in single‐cell transcriptomic data. To address this, the network was pruned in several steps to focus on biologically relevant interactions. First, meaningful genes such as differentially expressed genes (DEGs) and transition genes‐ were identified–those with significant changes along the pseudotime trajectory—by analyzing the time course data derived from the trajectory. Smoothing the single‐cell data was essential at this stage to mitigate stochastic noise inherent to single‐cell transcriptomics, enabling us to focus on global changes over time. Using smoothed data, Spearman correlation coefficients were calculated between genes to infer the directionality and signs of regulatory interactions. Positive correlations were assigned as activations, while negative correlations were assigned as inhibitions. A correlation cutoff (set to 0.7 in this study) was applied to remove weakly correlated interactions. The pruned network was then refined by removing terminal nodes—genes with an outdegree of zero—as these do not influence the overall dynamics of the network. This step significantly reduced the complexity of the network, focusing on regulatory components that shape system‐wide behavior. Next, strongly connected components (SCCs) within the network were connected, where every node is reachable from every other node within the SCC. SCCs play a critical role in shaping network dynamics as they represent feedback loops and core regulatory modules. The resulting subnetwork, composed of SCCs, was used to define the GRN structure for downstream dynamic modeling. Finally, Boolean regulation rules were determined for each regulatory interaction using binarized time‐course data. If a node lacks any activation input, self‐activation was added to maintain a potential regulatory edge. This finalized the GRN structure, integrating prior knowledge with trajectory‐specific data to capture the essential dynamics of the transition from normal to tumor states.

### Determining Boolean Regulation Rules of Genes

To determine Boolean regulation rules for genes, REVERT integrates the binarized expression data of genes with the structural information of the SCCs within the GRN. This approach captures the dynamic regulatory relationships between genes as cells transition along the pseudotime trajectory. The first step involves generating input–output pairs {(*I_t_*, *O*_*t* + *k*_)} from the pseudotime order, which represent the state of regulatory inputs at time *t* (*I_t_*) and the corresponding output states after a step size *k* (*O*_*t* + *k*_). To avoid any overlap between the input and output windows, the step size *k* was set larger than the width of the smoothing window applied to the pseudotime data (Figure S3a, Supporting Information). This ensures that input‐output pairs accurately reflect the causal relationships between gene expression states over time, mitigating any influence of stochastic noise in the single‐cell transcriptomic data. For each gene, a Boolean function *f* is identified such that it can represent the observed input–output relationships. To determine the best Boolean function for each gene, the score function for input–output pairs from the pseudotime order was implemented, as used in Pseudotime‐network‐inference.^[^ ^12^ ^]^ The score function *S*(*f*) evaluates the fitness of a Boolean function *f* to the input–output pairs

(2) $$S\;\left(f\right)=\sum_{t=1}^{m}S_{t}\left(f\right)$$

where *m* is the total number of input–output pairs, and *S_t_* (*f*) is defined as

(3) $$S_{t}\;\left(f\right)=\left\{\begin{array}{c} 1\\ 0 \end{array}\begin{array}{c} \;\mathrm{if}\;f\;\left(I_{t}\right)=O_{t+k}\\ \mathrm{otherwise}. \end{array}\right.$$

This scoring mechanism ensures that the Boolean function accurately captures the regulatory relationship between input states and the resulting output states. For genes with multiple input–output pairs, the function *f* with the highest score *S*(*f*) is selected as the most representative regulation. In cases where multiple Boolean functions achieve the same highest score, all such functions are retained. These are combined using a logical OR operation, reflecting the possibility of alternative regulatory mechanisms governing the gene expression dynamics. This redundancy preserves the complexity of regulatory interactions while maintaining the accuracy of the inferred GRN.

### Attractor Analysis

For a given Boolean regulation rules, dynamical state transition paths from random initial states will eventually converge to a few specific states, that is, the attractors of the Boolean network. The set of network states that converge to the same attractor is called the basin of attraction. Attractor analysis was conducted for each of the individually predicted networks using different combinations of hyper‐parameters including the smoothing window width and the step size in input–output pairs. For an ensemble of hyperparameters, the results revealed diverse cases where multiple attractors with small basin sizes coexist, or a single major attractor with a large basin size dominates. In the context of a transition state, the former case appears to be suitable, but the latter might be more appropriate if a trajectory from normal to cancer within the transition state is chosen during the pseudotime analysis. Boolean network simulations were performed using the BoolNet R‐package and synchronous update strategy. For a comprehensive understanding of the attractor distribution, REVERT provides quantified summaries encompassing the basin size of major attractor states, distance to normal and cancer attractors, the size of the resulting network, and the average agreement level, across a variety of hyper‐parameters. The average binarized gene expression vectors of cells within 20% from both ends of the pseudotime order were considered as normal/cancer cell attractor states, respectively.

### Computing the Distance to a Normal Attractor from each Attractor along the Normal‐Cancer Axis

Performing attractor analysis of a Boolean network reveals the attractor landscape, a discrete representation of the potential energy landscape of attractors and their basins. This landscape allows for the visualization of attractors and their basins by elucidating relationships between individual states in the state transition graph. To quantify attractor states in the context of malignancy, a virtual axis in the state space was presumed, connecting the normal attractor state to the cancer attractor state. The gene expression vector of an arbitrary state was then projected onto the axis to quantify its proximity to the normal attractor. This effective distance of a state vector, ***v***, in the state space was measured as the Euclidean distance between the normal attractor, ***v**_N_*, and the projection coordinates along the axis, ***v**_C_* − ***v**_N_*, as follows

(4) $$d=\frac{\left(v-v_{N}\right)\cdot \left(v_{C}-v_{N}\right)}{{\left|v_{C}-v_{N}\right|}^{2}}\;$$

When *d* approaches 0 or 1, it indicates that the state is close to either the normal or cancer attractor, respectively. The malignancy and basin size of each attractor state were determined by calculating the effective distance of the attractor and normalized counts from 1000 random initial states converging to the corresponding attractor.

### Defining Cancer Score by Quantifying the Attractor Landscape of a Boolean Network Model

To evaluate the degree of malignancy of a cell state, the attractor landscape was needed to be quantified so that not only the malignancy of attractor was compared but also the stability between attractors, i.e., their relative depth in the landscape. Perturbation analysis was used to compare the stability of different attractors by observing the uncertainty of steady states after the random perturbation on a state. A metric termed “attractor entropy” was defined to represent the uncertainty of the attractor state against the perturbation. A perturbed state within a basin of attraction can either return to the original attractor or hop into another attractor. The probability distribution of newly converged states following the perturbation was evaluated. The average entropy of attractor *i* is expressed as $S_{i}=\frac{\sum_{j}S_{i}^{j}}{N_{b}}$, where *j* denotes a state within the basin of attractor *i* and *N_b_* is the basin size. The entropy of state *j*, $S_{i}^{j}$ is given by $S_{i}^{j}=-\sum_{k}P_{k}\;\log_{2}P_{k}$ where *P_k_* represents the probability that the perturbed state converges to attractor *k*. The attractor entropy is maximal for a uniform distribution, specifically, $S_{max}=-\;\sum_{k}\frac{1}{N}\;\log_{2}\frac{1}{N}=\log_{2}N$, where *N* is the total number of attractors. Perturbation analysis was performed using the perturbNetwork function in the BoolNet R‐package. A single gene was randomly chosen and the output values of the corresponding Boolean functions were randomly permuted. The average entropy of each attractor corresponds to its depth in the attractor landscape, while the basin size represents the width of the attractor.

In the attractor landscape of a given cell state, the comprehensive evaluation of attractor valley depth, width, and their proximity to the cancer attractor collectively characterize the malignancy of the cell state. Consequently, a cancer score was introduced, which is conceptually expressed as the product of the amount of water filling the valleys in the landscape and how close these valleys are to the cancer attractor. This is calculated by summing the effective distance of each attractor along the n‐c axis multiplied by the area of each attractor valley across all attractors as follows

(5) $$\mathrm{Cancer}\;\mathrm{score}\;=\sum_{k}\left(1-\frac{S_{k}}{S_{max}}\right)\cdot \frac{B_{k}}{B_{N}+B_{x}+B_{C}}\cdot d_{k}$$

To generate the surface plot of the landscape, the persp function was used from the R graphic library. The contours of the valleys were modeled to fit to the function $f\left(x\right)∼-Se^{-\frac{x^{2}}{B^{2}}}$.

### Predicting the Cancer Reversion Targets through Systematic In Silico Gene Perturbations

The construction of an executable logical GRN enables simulations of gene perturbations and predictions of their impact on the overall network, thereby facilitating the identification of potential therapeutic targets. The network was initially perturbed by manipulating gene expression, fixing it either to 0 (knockdown) or 1 (overexpression). The attractor landscape changes due to the perturbation, consequently resulting in alterations to the cancer score. The optimal target gene would be the one that induces the most substantial reduction in the cancer score. Our analysis was limited to instances where the initial cancer score of the transition state exceeds 0.5, and considered as successful cancer reversion when the final cancer score is 0.1 or less. In complicated networks, however, single‐gene perturbation alone might be insufficient to achieve a final cancer score below 0.1. Double‐gene perturbation was then examined using combinations of the top 5 genes identified as effective in single‐node perturbations. The fixGenes function in the BoolNet library was used for node fixation. Finally, the most effective combination of two candidate genes was mapped onto the regulon network by SCENIC, a subnetwork composed of TFs and their target genes, derived from scRNA‐seq of the transition state. Common target genes of the two TFs were then extracted as the ultimate candidate genes for cancer reversion.

### USP7 Inhibitor Treatment to the Patient‐Derived Colon Cancer Organoid

The cultured organoids were treated with 5, 10, 15, 20 µM of the USP7 inhibitor P22077 (#S7133, Selleck Chemicals, USA) or vehicle (DMSO; Sigma‐Aldrich, USA) for 10 days. Medium containing P22077 was replaced daily.

### Organoid Size Measurement using OrganoID

Images of organoids within Matrigel droplets were captured at three to four locations within the gel and at two to three different height layers using a microscope equipped with a 4x objective on days 0, 5, and 10 of culture. The images were analyzed using OrganoID,^[^ ^50^ ^]^ applying a hyperparameter set with a threshold of 0.2, edge sigma of 0.4, minimum edge of 0.01, maximum edge of 0.1, and a minimum area of 50 px. The areas measured in pixels were converted to square micrometers (µm^2^) using a coefficient estimated by ImageJ.

### RNA Isolation from the Organoids

Organoids embedded in Matrigel were harvested by centrifugation for 5 minutes at 500 x g at 4°C. Matrigel was discarded, and the organoids were washed twice with DPBS. RNA was extracted using an RNA‐spin kit (INTRON).

### Bulk RNA‐seq Library Construction of the Organoids

Total RNA was extracted from the collected cell or tissue samples using the RNA extraction kit (Intron, Korea) following the manufacturer's instructions. RNA integrity was assessed using the Agilent 2100 Bioanalyzer (Agilent Technologies), ensuring all samples had an RNA Integrity Number (RIN) greater than 7. RNA concentration was measured using the Qubit RNA Assay Kit (Thermo Fisher Scientific). RNA sequencing libraries were constructed using the TruSeq RNA Library Prep Kit v2 according to the manufacturer's protocol. Briefly, mRNA was isolated from total RNA using poly‐T oligo‐attached magnetic beads and fragmented into small pieces under elevated temperature. The first strand cDNA was synthesized using random hexamer primers and reverse transcriptase, followed by synthesizing the second strand cDNA using DNA Polymerase I and RNase H. The cDNA fragments underwent end repair, A‐tailing, and ligation to adapters. The products were then purified and enriched with PCR amplification to create the final cDNA library. The quality and quantity of the libraries were assessed using the Agilent 2100 Bioanalyzer and the Qubit DNA Assay Kit. Libraries were then sequenced on the Illumina Hi‐Seq 2500 to generate paired‐end reads of 101 base pairs.

### Alignment and Preprocessing of Bulk RNA‐seq Data of Colon Cancer Organoids

Sequencing libraries were prepared using TruSeq RNA Sample Preparation kit v2 (Illumina Inc., USA). After pooled libraries were denatured, each library was sequenced using the 100 bp paired‐end mode of the TruSeq Rapid PE Cluster Kit and TruSeq Rapid SBS Kit with HiSeq 2500 (Illumina Inc., USA). To quantify the total RNA of colon cancer organoids, sequencing libraries were prepared using TruSeq RNA Sample Preparation Kit v2. After pooled libraries were denatured, each library was sequenced using the 100 bp paired‐end mode of the TruSeq Rapid PE Cluster Kit and TruSeq Rapid SBS Kit with HiSeq2500 (Illumina Inc.). The prepared RNA‐seq data were trimmed using Trimmomatic^[^ ^51^ ^]^ version 0.39. The trimmed reads were aligned to the mm10 reference genome using STAR version 2.7.7a with the default parameter. The mapped reads were indexed and sorted by samtools version 1.7. Then HTSeq^[^ ^52^ ^]^ version.0.12.4 was used to quantify read coverage per gene. For all human RNA seq data, the alignment pipeline (Trimmomatic – STAR – HTSeq) with hg38 reference genome was also performed. Next, batch‐effect corrections were performed by ComBat‐seq.^[^ ^53^ ^]^

### GSEA Analysis using the fgsea R Package

Gene Set Enrichment Analysis (GSEA) was performed to identify significantly enriched pathways in our bulk RNA‐seq data using the fgsea R package.^[^ ^54^ ^]^ Differential gene expression analysis was first conducted using DESeq2,^[^ ^55^ ^]^ generating a ranked list of genes based on log2 fold changes between experimental and control groups. Using the fgsea package, the analysis with the following parameters was performed: a minimum gene set size of 15, a maximum gene set size of 500, and 1000 permutations to assess statistical significance. The results were filtered to include only pathways with a nominal *p*‐value of less than 0.05. Significant pathways were further visualized to interpret the results. Enrichment plots for key pathways were generated to illustrate the distribution of genes within these pathways and their contribution to the enrichment score.

## Conflict of Interest

The authors declare no conflict of interest.

## Author Contributions

K.‐H.C. designed the project and supervised the research. D.S. and J.‐R.G. performed the modeling and analysis. J.‐R.G. and S.J. performed in vitro experiments. Y.C., H.‐P.K., and T.‐Y.K. established paired normal colon epithelium and colon cancer organoids. D.S., J.‐R.G. and K.‐H.C. wrote the manuscript.

## Supporting information



Supporting Information

Supplemental Table 1

Supplemental Table 2

Supplemental Table 3

## Data Availability

All original sequencing data are publicly available on the NCBI Gene Expression Omnibus (GEO) (https://www.ncbi.nlm.nih.gov/geo/). The accession number for single‐cell RNA sequencing data reported in this paper is available on the GEO database under the accession codes such as GSE283167 (Super‐series). Correspondence and requests for materials should be addressed to Kwang‐Hyun Cho (ckh@kaist.ac.kr). The authors declare that all the data supporting the results of this study are available within the article and its supplementary data files from the corresponding author upon reasonable request. The script used in the construction of a dynamic network model is publicly available at https://github.com/dshin‐ncc/REVERT.
