# Supplementary material for: Attractor Landscape Analysis Reveals a Reversion Switch in the Transition of Colorectal Tumorigenesis
Source: Adv Sci (Weinh). 2025 Jan 22;12(8):2412503. doi: 10.1002/advs.202412503 (PMC11848608; doi:10.1002/advs.202412503)
Supplement: Supplementary file 1 — Supporting Information [file ADVS-12-2412503-s002.pdf]

ADVANCED SCIENCE
Open Access

Supporting Information

for *Adv. Sci.*, DOI 10.1002/advs.202412503

Attractor Landscape Analysis Reveals a Reversion Switch in the Transition of Colorectal Tumorigenesis

*Dongkwan Shin, Jeong-Ryeol Gong, Seoyoon D. Jeong, Youngwon Cho, Hwang-Phill Kim, Tae-You Kim and Kwang-Hyun Cho**

Supporting Information

Attractor Landscape Analysis Reveals a Reversion Switch in the Transition of Colorectal Tumorigenesis

*Dongkwan Shin, Jeong-Ryeol Gong, Seoyoon D. Jeong, Youngwon Cho, Hwang-Phill Kim, Tae-You Kim, and Kwang-Hyun Cho**

Figure S1- S9

Table S1- S3

Supplementary Figures

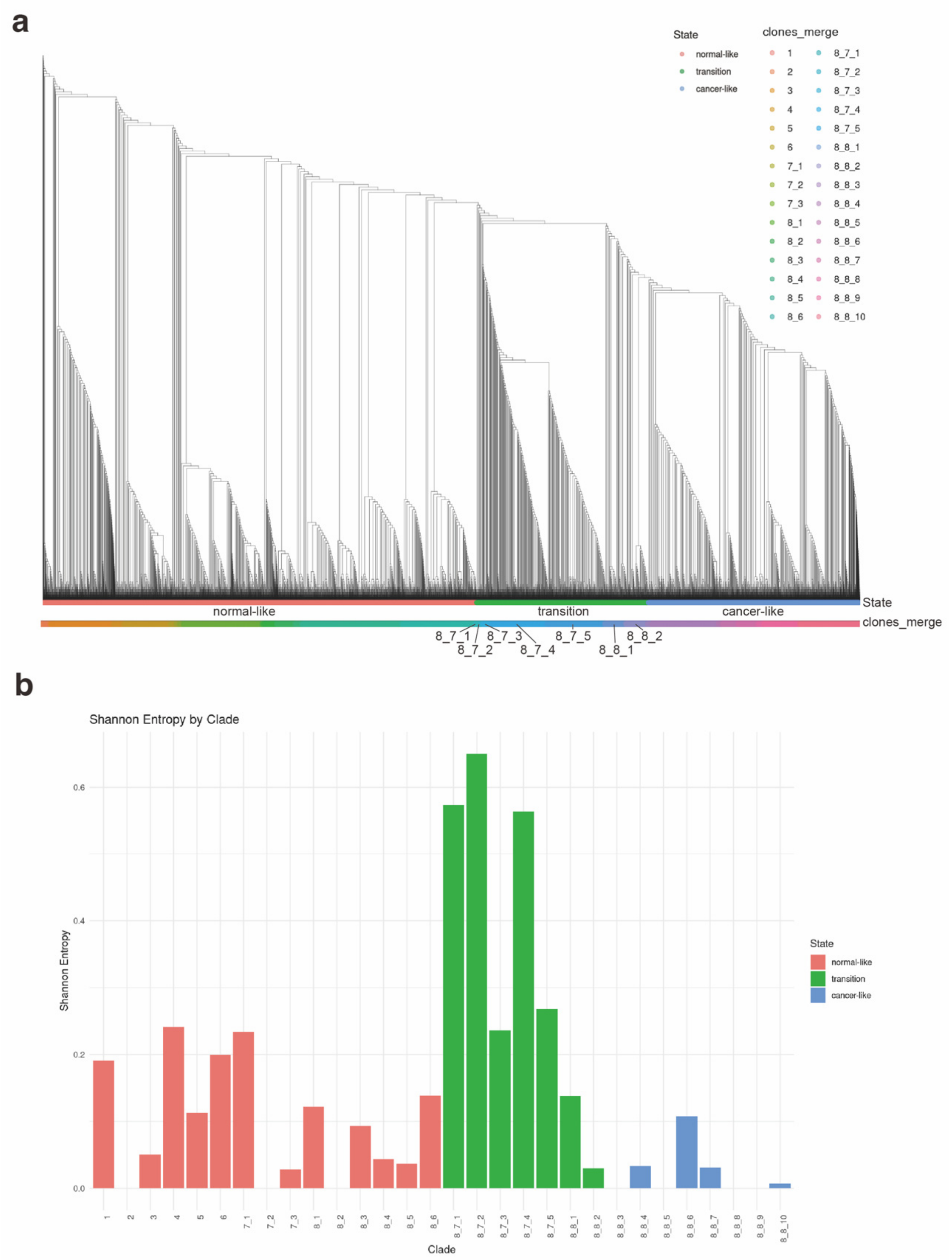

Figure S1. Identification of the transition state in tumorigenesis through phylogenetic tree analysis. (a) Phylogenetic tree constructed based on inferred copy number variations (CNVs) to capture the evolutionary relationships among single cells. The tree was divided into 30 smaller clades, enabling the analysis of heterogeneity within each clade. (b) Quantification of clade heterogeneity using Shannon entropy. The transition state was

identified as the region within the tree where Shannon entropy exhibited a marked increase, indicating a significant mixture of normal and tumorigenic cell populations.

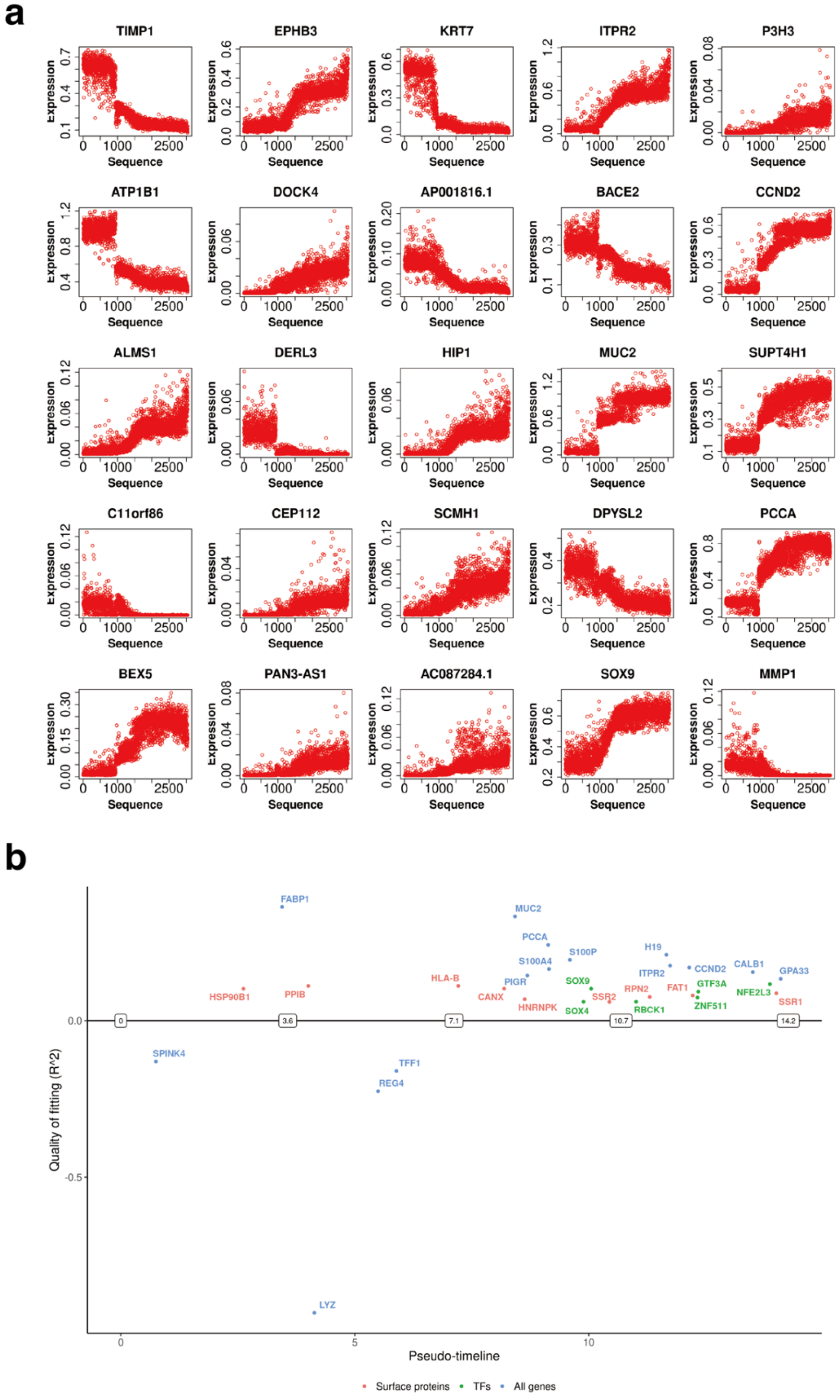

Figure S2. Genes highly relevant to the pseudotime trajectory. (a) Temporal profiles of the top 25 differentially expressed genes (DEGs) highly correlated with pseudotime. (b) Visualization of transition genes along the pseudotime trajectory. The figure was generated

using Geneswitches (see Methods). Genes that are switched on are plotted above the line, while genes that are switched off are plotted below the line.

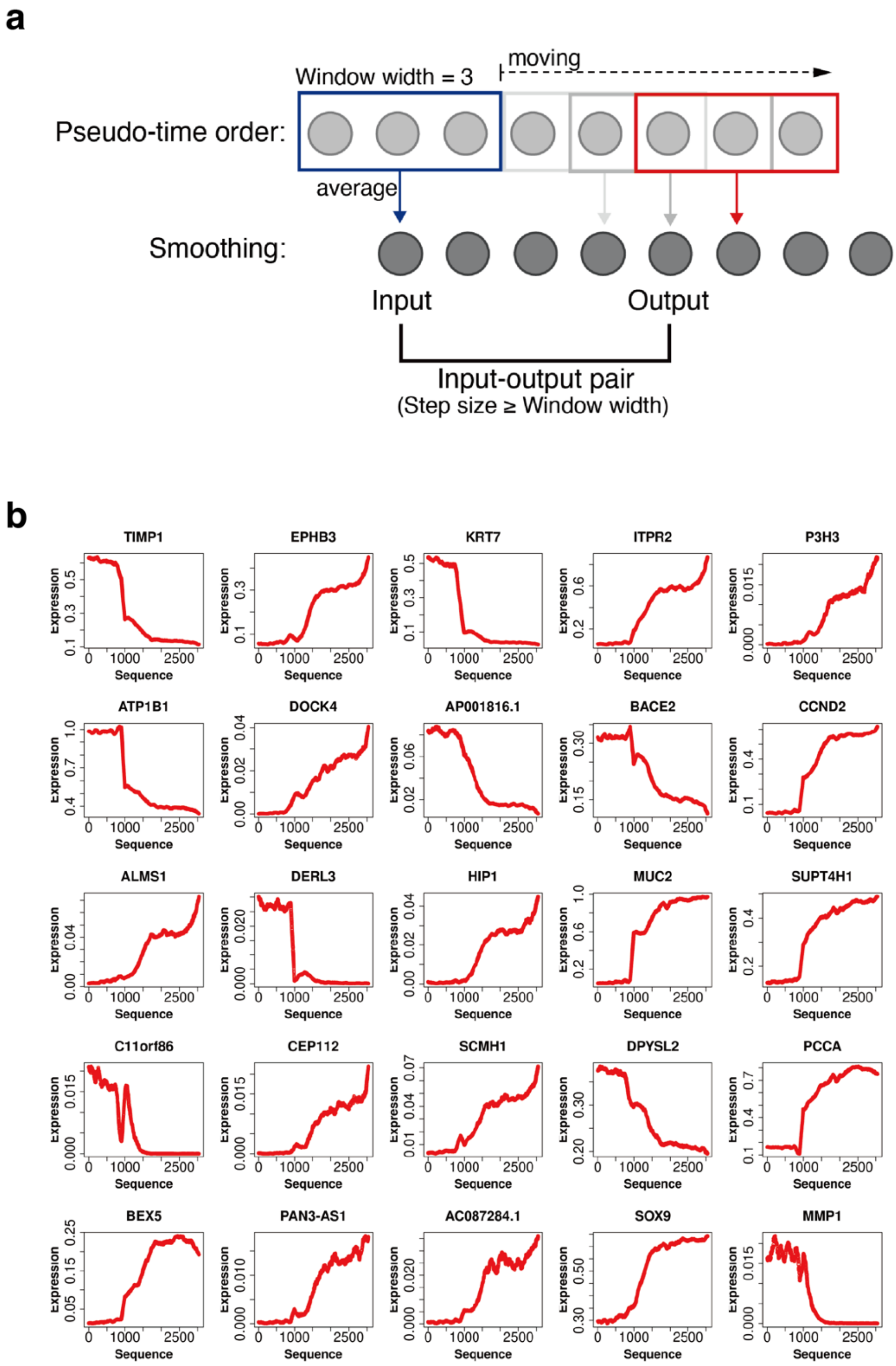

Figure S3. Smoothing of temporal gene expression profiles using a moving window approach. (a) Schematic illustration of the smoothing procedure. The step size was set larger than the width of the smoothing window to prevent overlap between input and output windows. (b) Smoothed temporal profiles for the top 25 differentially expressed genes (DEGs) highly correlated with pseudotime, obtained through the moving window smoothing process.

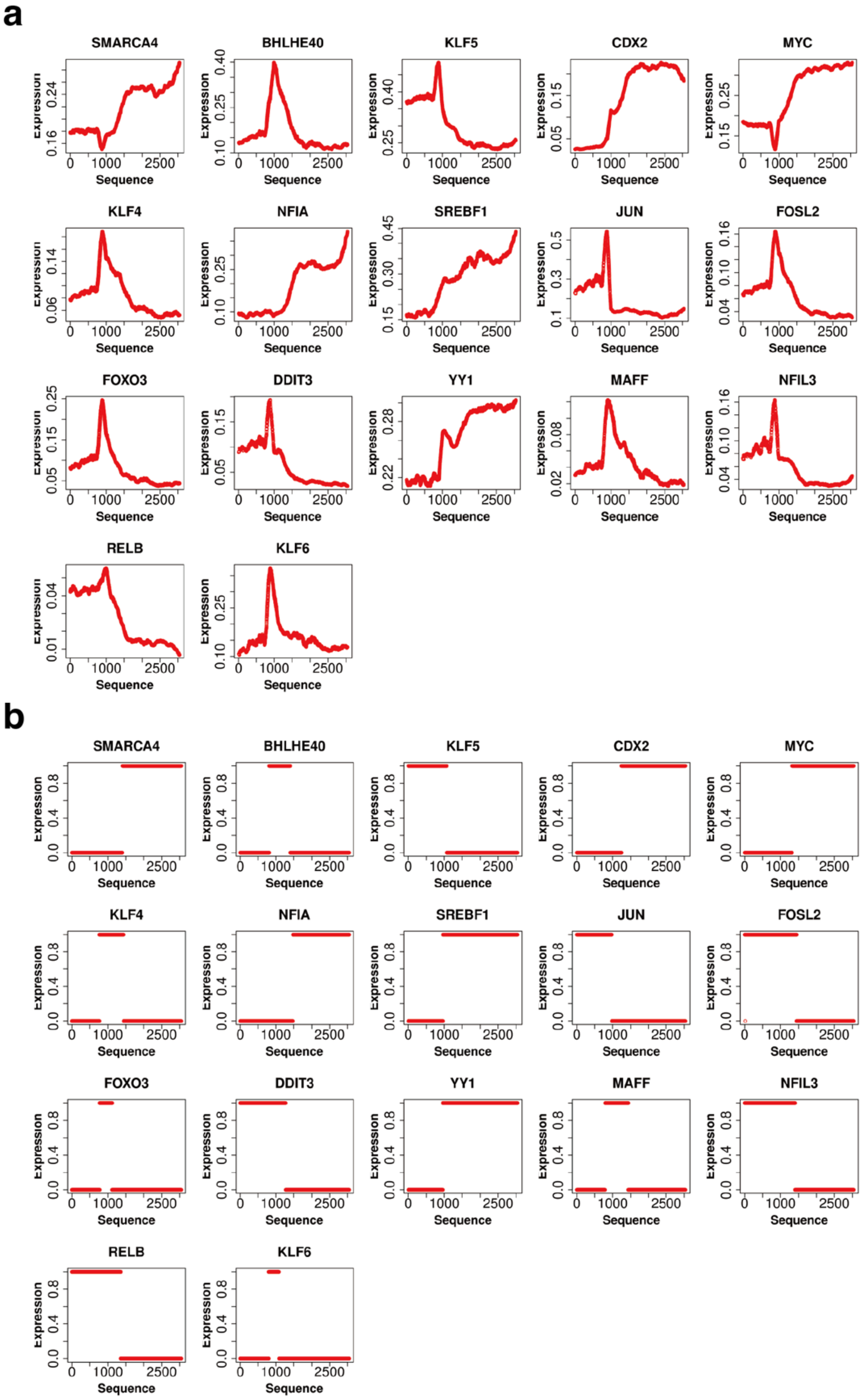

Figure S4. Temporal profiles of smoothed (a) and binarized (b) gene expression in the strongly connected component (SCC).

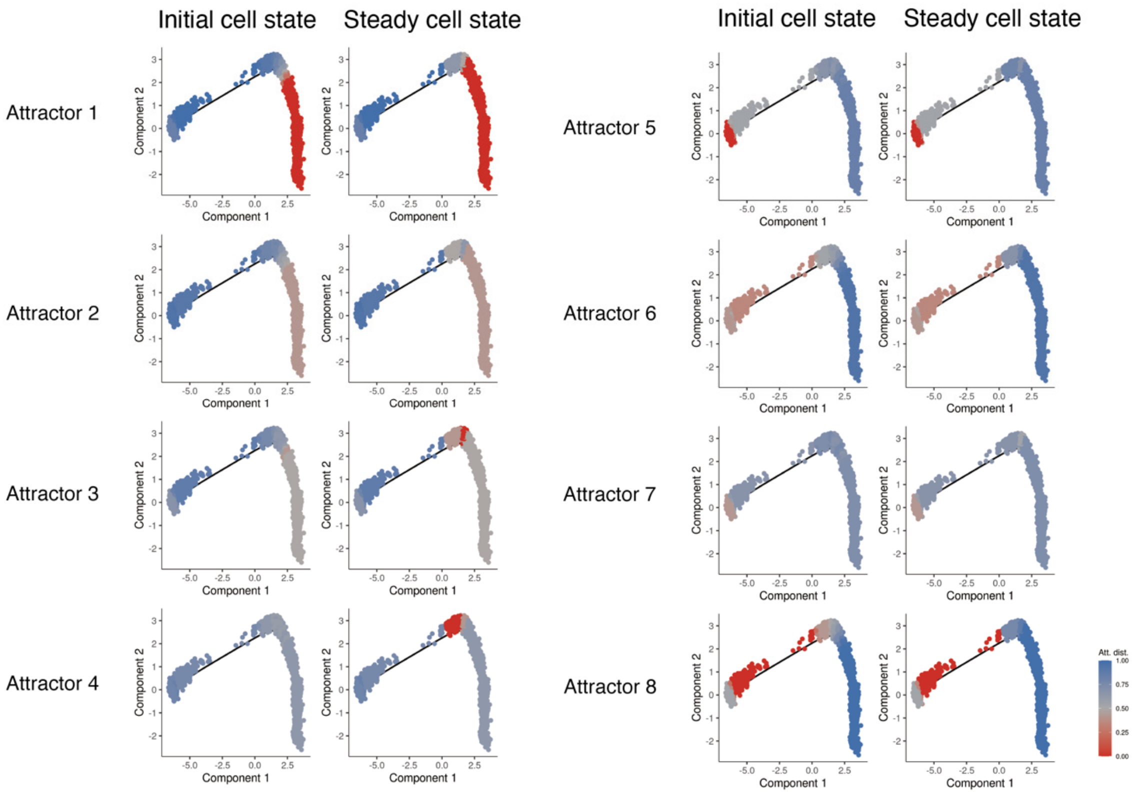

Figure S5. Euclidean distance of initial states or steady states of single cells to each attractor. Initial cell states represent the binarized expression level vector of each cell, while steady cell states indicate the binarized expression level vectors to which the system ultimately converges according to Boolean functions. The color bar represents the distance of each cell state from the corresponding attractor. Red indicates cell states closer to the attractor, reflecting greater similarity, while blue represents states further away.

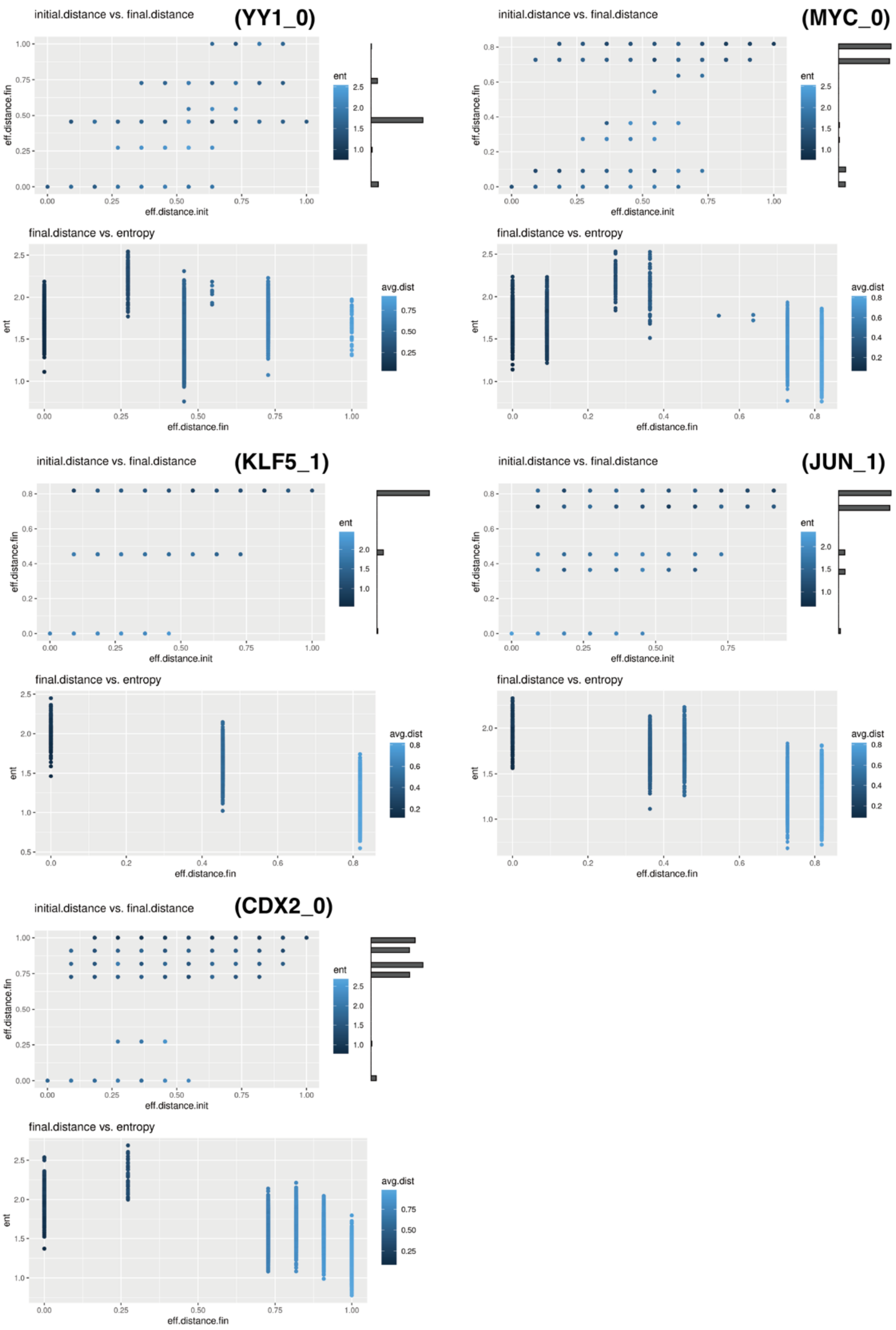

Figure S6. Basin size and attractor entropy following perturbation of a specific gene. Attractors were derived from 10,000 random initial states. The effective distances of the attractors are represented on the y-axis, while the effective distances of the initial states are shown on the x-axis. The histogram on the y-axis indicates the basin size of the attractors.

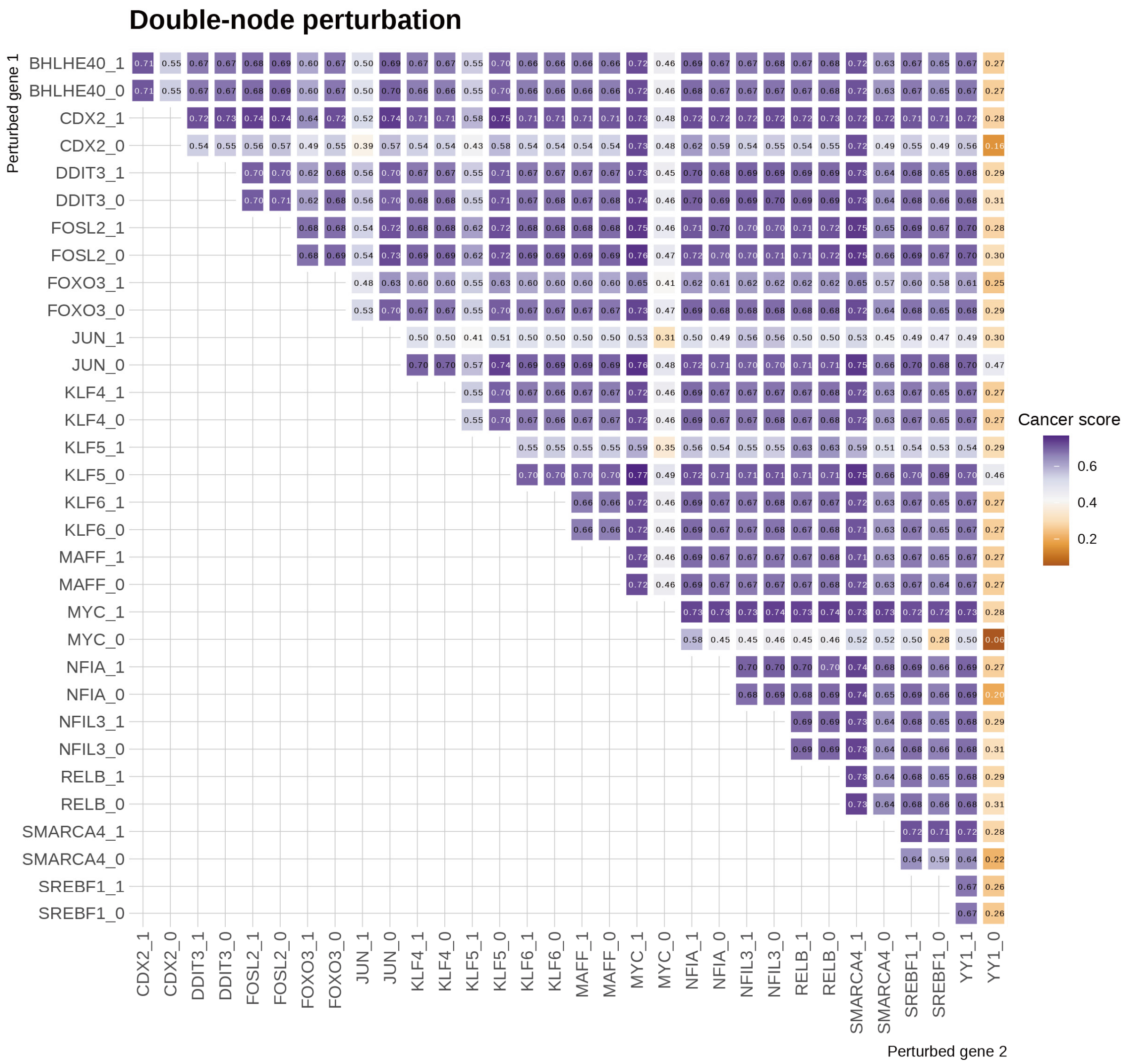

Figure S7. Double-node perturbation analysis for identifying therapeutic targets for cancer reversion. Each score represents the cancer score calculated from the attractor landscape following perturbation of the corresponding pair of genes.

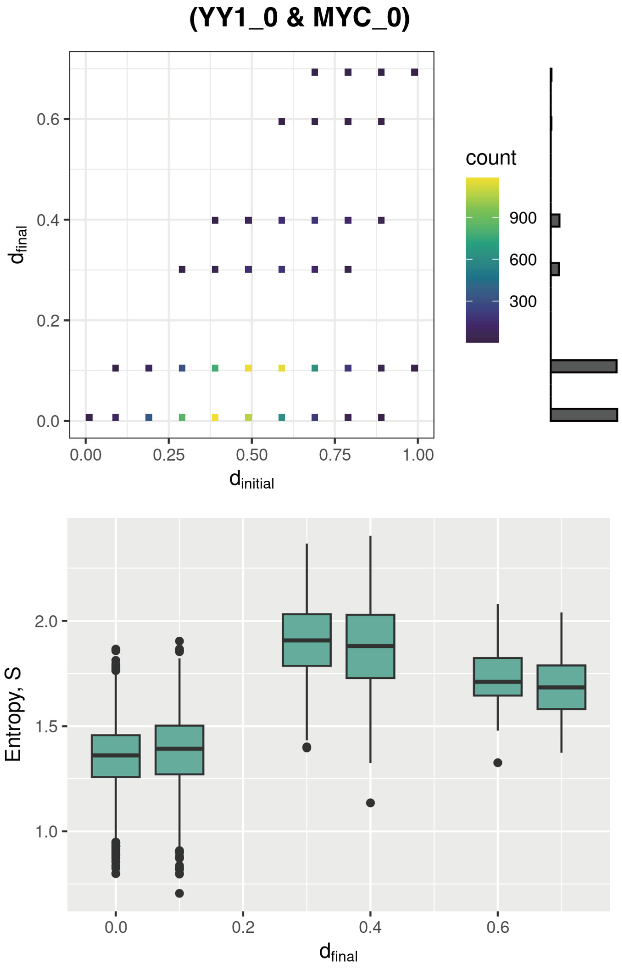

Figure S8. Basin size (top) and attractor entropy (bottom) following the knockout perturbations of YY1 and MYC. The double knockout of YY1 and MYC resulted in the disappearance of the cancer attractor and the emergence of dominant normal and near-normal attractors in the landscape.

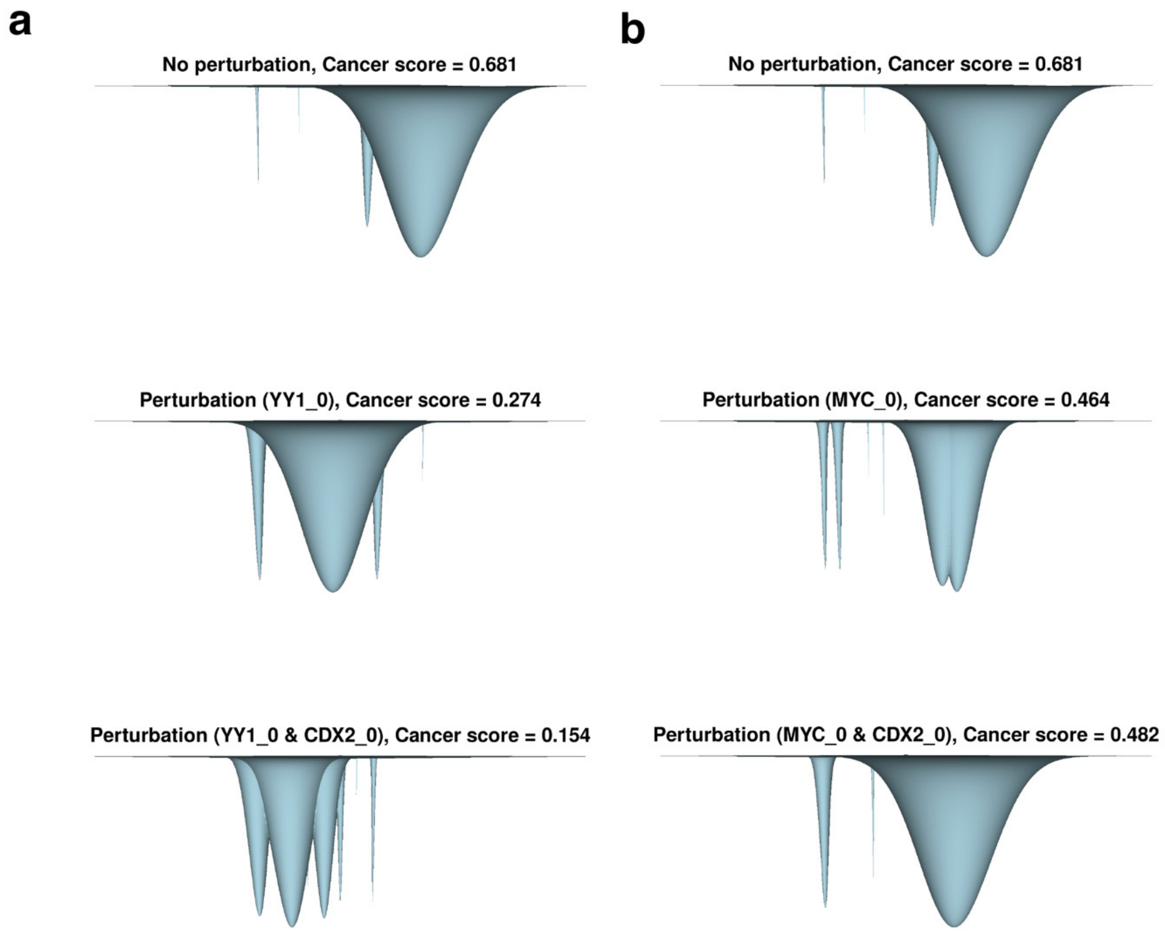

Figure S9. Changes in the attractor landscape following double-node perturbations of YY1 and CDX2 (a) and MYC and CDX2 (b). The double knockout of YY1 and CDX2 is the second most effective strategy, whereas targeting MYC and CDX2 results in a less favorable outcome compared to targeting MYC alone.

Supplementary Tables

Table S1: Summary of all the Boolean functions of the network genes inferred by REVERT. (Separated csv file: TableS1.csv)

Table S2: Boolean functions combined using the logical OR operation and agreement level of the resulting input-output pairs with scRNA-seq data. (Separated csv file: TableS2.csv)

Table S3: Summary of double-node perturbations across various hyper-parameter sets. Highly effective cases with cancer score after perturbations less than 0.15 are summarized. PerturbedGene1 and PerturbedGene2 represent the two perturbed genes. FinalCS, InitialCS, and DiffCS indicate the final cancer score and initial cancer scores, and the difference between them, respectively. SmoothWidth, Tstep, and iDEG are hyper-parameters for determining a network model, representing the width of the smoothing window, the time step size for input-output pairs, and the number of DEGs, respectively. REVERT summarize six features for the dynamic network model: NetSize (size of the constructed network), AgreeLevel (averaged agreement level of all genes), MeanTopAtt (mean activity of gene expressions in the largest attractor), BasinSize (basin size of the largest attractor), Distance2Normal (minimal distance to normal attractor among all attractors), and Distance2Cancer (minimal distance to cancer attractor among all attractors). (Separated csv file: TableS3.csv)
